# Comparing statistical analyses to estimate thresholds in ecotoxicology

**DOI:** 10.1371/journal.pone.0231149

**Published:** 2020-04-08

**Authors:** Marcos Krull

**Affiliations:** Department of Aquatic Health Sciences, College of William and Mary, Virginia Institute of Marine Science, Gloucester Point, Virginia, United States of America; Universidad Rey Juan Carlos, SPAIN

## Abstract

Different methods are used in ecotoxicology to estimate thresholds in survival data. This paper uses Monte Carlo simulations to evaluate the accuracy of three methods (maximum likelihood (MLE) and Markov Chain Monte Carlo estimates (Bayesian) of the no-effect concentration (NEC) model and Piecewise regression) in estimating true and apparent thresholds in survival experiments with datasets having different slopes, background mortalities, and experimental designs. Datasets were generated with models that include a threshold parameter (NEC) or not (log-logistic). Accuracy was estimated using root-mean square errors (RMSEs), and RMSE ratios were used to estimate the relative improvement in accuracy by each design and method. All methods had poor performances in shallow and intermediate curves, and accuracy increased with the slope of the curve. The EC5 was generally the most accurate method to estimate true and apparent thresholds, except for steep curves with a true threshold. In that case, the EC5 underestimated the threshold, and MLE and Bayesian estimates were more accurate. In most cases, information criteria weights did not provide strong evidence in support of the true model, suggesting that identifying the true model is a difficult task. Piecewise regression was the only method where the information criteria weights had high support for the threshold model; however, the rate of spurious threshold model selection was also high. Even though thresholds are an attractive concept from a regulatory and practical point of view, threshold estimates, under the experimental conditions evaluated in this work, should be carefully used in survival analysis or when there are any biological reasons to support the existence of a threshold.

## Introduction

The existence of thresholds in ecotoxicology has been questioned and addressed for more than fifty years now [1–4]. For many years, precise and accurate estimation of thresholds was impractical, unreliable or too complex to be done with the available tools and methods. Statistical methods such as analysis of variance and generalized linear models (GLM), used to estimate the no observed effect concentration (NOEC) and the effect concentration (ECx) respectively, were the best available tools to analyze ecotoxicological data. Both analysis of variance and GLM remain as the most used approaches in the field. However, serious criticisms have been made of both metrics (e.g., [5]) and ecotoxicologists are now suggesting that thresholds estimates are more ecological relevant meaningful and more useful in risk assessment [6–8].

With computational advances, different statistical methods have been developed and applied in ecotoxicology (e.g., [4, 9–11]). One of the first recognized and most used approaches is the no-effect concentration (NEC) model [3]. The term NEC now seems to describe a series of models with a threshold parameter that can be assumed to be time-independent and have an elimination rate of the organism as a parameter (e.g. [7]) or not (e.g. [9, 10]). Another common approach used in ecotoxicology is the piecewise regression, which also includes a threshold parameter (e.g., [11]). The term EC0 have also been used to describe thresholds (e.g., [11]) and by definition, both terms, NEC and the EC0, assume that there is no effect before the threshold concentration other than background mortality.

Previous simulation studies have shown that the NEC models can be used to accurately estimate thresholds in survival, time to death and count data (e.g. [7,9]). However, given that different methods are now available, the question remains about which statistical method is the most accurate to estimate thresholds in ecotoxicology. Another important question is how these models behave with datasets for which a true threshold does not exist, or what is the rate of spurious threshold detection. In many situations, models with and without thresholds could fit to the observed data equally well and deciding which model to use is not straightforward [12]. In such cases, an apparent threshold can be estimated, which can have practical value [13]. However, the rates of spurious threshold estimation and the advantages of estimating an apparent threshold instead of estimating lower ECx values have not been fully accessed yet. At the same time, misspecification of the correct model may also result in biased ECx estimates in datasets that have a threshold.

This paper aims to (i) identify the most accurate and precise method to estimate critical thresholds in survival data among three different methods, (ii) compare the accuracy of 2 different sampling designs in estimating threshold, (iii) evaluate the rate of spurious threshold detection when using model selection, (iv) evaluate if there are any advantages in estimating an apparent threshold from datasets that do not have a true threshold, and (v) evaluate the implications of specifying the incorrect model (i.e., with or without a threshold) when estimating ECx values. Three different statistical methods were selected to estimate thresholds in survival datasets: (i) maximum likelihood estimation of the threshold parameter by fitting a NEC model; (ii) a Bayesian estimation of the NEC model using Markov Chain Monte Carlo (MCMC) methods, and (iii) generalized linear Piecewise regression.

## Methods

### Simulated data

All datasets were generated to simulate the survival of ten organisms exposed to different effluent concentrations. A non-linear model with a threshold parameter was modified from Pires et al., [9] and used by Fox [10], to describe the survival probability of organisms exposed to an effluent with the equation

$$p_{i}=le^{\left[-m\left(x_{i}-c\right)I\left(x_{i}-c\right)\right]},$$ (1)

$$I\left(x_{i}-c\right)=\{\begin{array}{c} 1,x>c\\ 0,x\leq c \end{array}$$

where (i) *p*_*i*_ is the survival probability in the *x*_*i*_ concentration, (ii) *l* is the intercept, or the survival probability when the effluent concentration is equal to 0, (iii) *m* is the rate of decay (throughout the paper, *m* is referred as a “slope” to make it consistent with all the other models), (iv) *c* is the threshold parameter, and (v) *I*(*x*_*i*_−*c*) is the indicator function. When *x*_*i*_ is lower or equal to the threshold, the probability of survival is equal to the intercept.

**Fig 1:**
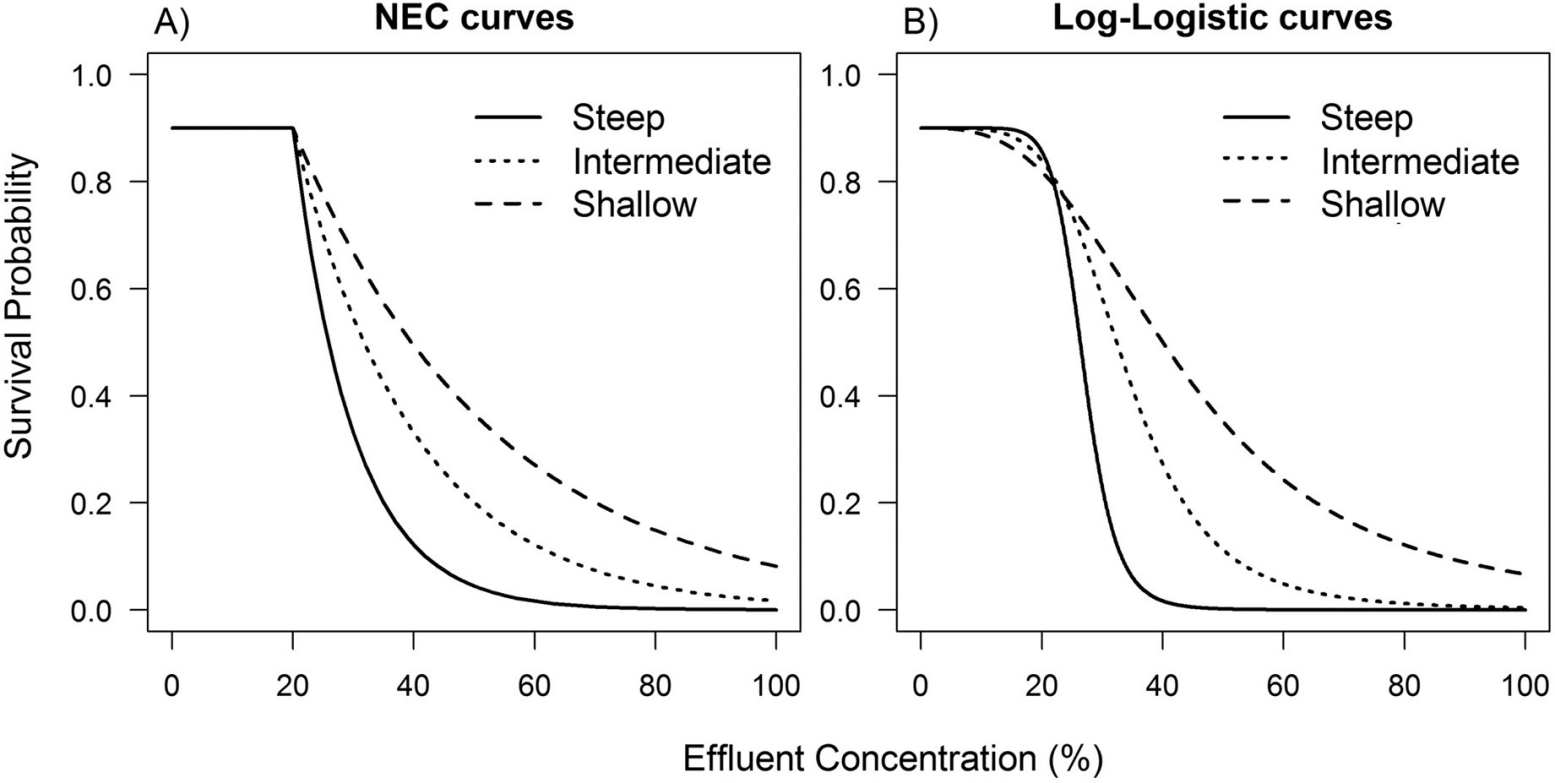
Three types of probability curves with medium background mortality. (a) NEC and (b) log-logistic models used in this study.

Three types of curves were generated with different slopes parameters: (i) shallow (*m* = 3), (ii) intermediate (*m* = 5) and (iii) steep (*m* = 10) slopes (Fig 1A). In all curve, the threshold concentration was set to 20% of the effluent. This concentration was selected to make sure that most datasets, within all different types of curves, designs and background mortalities, would have at least one concentration with partial kills (i.e., mortality is higher than 0 and lower than 100% at that concentration). The application of the methods described in this manuscript is not recommended for datasets without partial kills and, for this reason, simulations with different values of the threshold parameter were not conducted.

Three different intercepts were selected to consider different levels of background mortality: the lowest (0.95), medium (0.90) and the highest (0.85) background mortality. The rates of test rejection of all background mortalities are presented in S1 Table in S1 Appendix. Datasets that had the mean survival lower than 80% in the control were discarded and replaced by another dataset. Note that as background mortality increases, variability around the threshold should also increase.

Datasets without the presence of a true threshold were generated using a three parameter log logistic model as described by Ritz [14]

$$p_{i}=\frac{d}{1+e^{\left(b(\log(x_{i})-\log(e)\right)}},$$ (2)

where (i) *p*_*i*_ is the survival probability in the *x*_*i*_ concentration, (ii) *b* is the slope, (iii) *e* is the inflection point, or the EC50, and (iv) *d* is the intercept. Three types of curves were created with the same slope parameters of the NEC models (i.e. 3, 5 and 10 for the shallow, intermediate and steep curve respectively). The EC50 of the NEC models were used as the parameter *e* for each type of curve in order to generate similar curves among the two models (Fig 1B). Eqs (1) and (2) were solved to estimate the true EC5, EC10 and EC50 values from the probability curves. The true values of the EC50, EC10, and EC5 for both types of curves are presented in Table 1.

**Table 1:** True values of ECx and threshold for the NEC and log-logistic curves.

	NEC curve	Log-logistic curve
**Shallow (Slope = 3)**		
EC50	43.10%	43.10%
EC10	23.51%	20.72%
EC5	21.71%	16.15%
Threshold	20.00%	-
**Intermediate (Slope = 5)**		
EC50	33.86%	33.86%
EC10	22.10%	21.82%
EC5	21.02%	18.79%
Threshold	20.00%	-
**Steep (Slope = 10)**		
EC50	26.93%	26.93%
EC10	21.06%	21.61%
EC5	20.50%	20.06%
Threshold	20.00%	-

### Experimental designs

**Fig 2:**
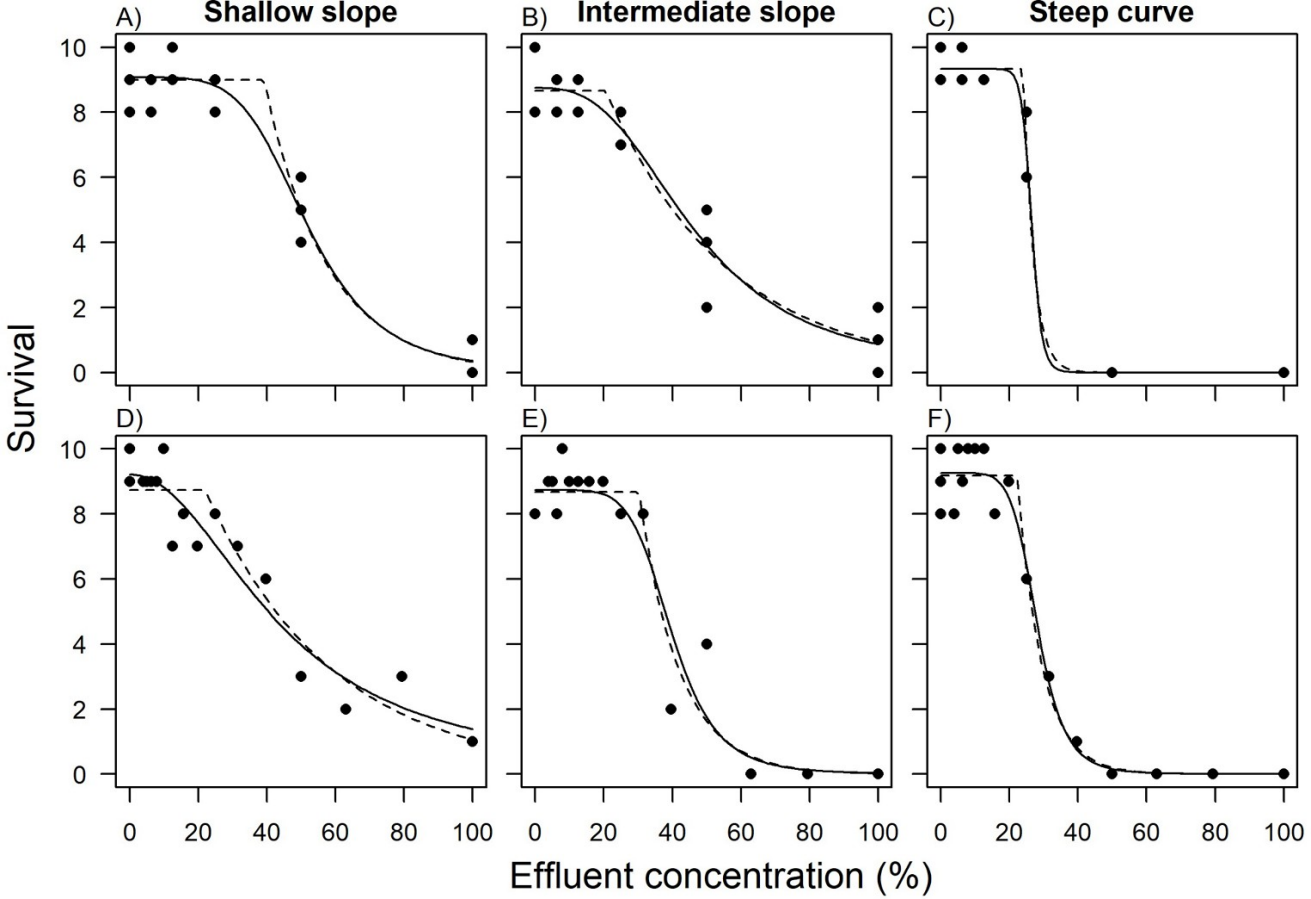
Examples of simulated datasets with medium background mortality for each slope. (a, b, c) categorical and (d, e, f) continuous design. Solid and dashed lines represent the three parameter log logistic and MLE NEC models respectively.

Two different designs were used: (i) the categorical design, which consisted of 5 concentrations with 3 replicates per concentration plus the control (i.e. 0%, 6.25%, 12.5%, 25%, 50% and 100%) and (ii) the continuous design, without replicates at the same treatment and fifteen concentrations equally spaced in the log_e_ scale from 100% to 3.94%. Please notice that even though effluent concentrations are presented as percentage throughout this work, concentrations could also be expressed as 0, 0.625, 0.125, 0.25, 0.5 and 1 μg/L (or mg/L) of a hypothetical chemical without loss of generality. The categorical design follows similar procedures recommended by the EPA for measuring the acute toxicity of effluents [15]. In the continuous design, replication only occurred in the control treatment to ensure quality control of the organisms used in the test. Thus, both designs had the same number of experimental units (n = 18) and number of organisms (n = 180). Each concentration is assumed to be independent (true replicates). The concentrations in the continuous design include the same 5 concentrations of the categorical design plus 10 different concentrations. For each design (i.e., categorical and continuous), background mortality (i.e., low, medium and high), slope (i.e., shallow, intermediate and steep) and type of dataset (with and without a threshold parameter), one thousand datasets were generated. As a result, 36 thousand datasets were analyzed. One example of each slope and design for the datasets generated with the NEC model is provided in Fig 2.

### ECx estimates

For each dataset, three parameter log logistic models were fit to estimate the EC5, EC10 and EC50 values. To allow comparisons among all types of curves and designs, and for the simplicity of this work, the same model was fit to all datasets even though in many cases, three parameter Weibull models were selected as the best fit based on the Akaike’s information criterion (AIC). All models were fitted using maximum likelihood estimates (MLE) with the quasi-Newton method, and the confidence intervals were estimated using the Delta method, with the drc R package [16]. Because it would be unfeasible to manually evaluate model fit for thousands of models, such as checking the residuals distribution and Q-Q plots, wide confidence intervals were used as a proxy for very poor model fit. Indeed, after a closer inspection of these models and datasets, models with very wide confidence intervals presented very poor model fit and should not be used for statistical inference. Therefore, models with wide confidence intervals (i.e. above 100) were counted and excluded from the analysis. This approach was used for all models fitted in this manuscript.

### MLE of the NEC

This approach consists in fitting the generalized nonlinear model described in Eq 1 using MLE (quasi-Newton method) of the parameters. Because background mortality was added to all types of curves, a three-parameter model (i.e., intercept, slope and threshold parameter) was used. Confidence intervals were estimated using the Delta method and models were fit with the drc R package [16].

### Bayesian NEC

The model described in Eq 1 was fit using Markov Chain Monte Carlo (MCMC) methods with a Gibbs sampler algorithm. Uniform distributions were used as uninformative flat priors for both the intercept and slope parameters, assuming a minimum and maximum value of 0 and 1, and 0 and 20 for the intercept and slope, respectively. Uninformative priors were also used for the threshold parameters using a gamma distribution with the shape and scale parameter equal to 0.001. Three independent chains were used in parallel with 20^5^ iterations for adaptation. An additional 10^5^ iterations were run and the samples were monitored every 10 steps. The Bayesian models were fitted with the rjags R package [17].

### Piecewise regression

The generalized linear piecewise regression, using a logit link function, can be written as

$$logit\left(\pi_{i}\right)=\beta_{0}+\beta_{1}x_{i}+\beta_{2}\left(x_{i}-\Psi \right)I\left(x_{i}-\Psi \right),$$ (3)

$$I\left(x_{i}-\Psi \right)=\{\begin{array}{c} 1,x>\Psi \\ 0,x\leq \Psi \end{array}$$

where (i) *β*_*0*_ is the intercept, (ii) *β*_*1*_ is the first slope on the left, (iii) *β*_*2*_ is the difference-in-slopes after the threshold, (iv) *ψ* is the threshold parameter and (v) *I*(*x*_*i*_-*ψ)* is the indicator function, similar to the indicator function in Eq (1). The first slope was set to zero, so the model has three parameters and it assumes that there is no effect before the threshold. Note that different from the other approaches, this model can also include more than one threshold. The GLM models were fit using a bias-reduction method [18] to avoid perfect separation, or monotone likelihood, where nonfinite estimates of coefficients or standard errors are produced [19]. Without the bias correction many of the models produced extremely high or infinite standard errors that would have to be excluded from the analysis. To avoid convergence to local minima, four different initial values for the threshold concentration were used (i.e., 10%, 15%, 20% and 25%). GLM models were fit using the brglm package [20]. Piecewise regressions models were fit to the logit GLM models with the Segmented R Package [21].

### Data analysis

For each method, accuracy was estimated with the root-mean-square error (RMSE) as

$$RMSE=\sqrt{\frac{1}{n}\sum_{i=1}^{n}{\left(\delta_{i}-\theta \right)}^{2},}$$ (4)

Where *δ*_*i*_ is the ith parameter estimate and *θ* is the true parameter value. Thus, smaller RMSE values indicate higher accuracy. Note that the RMSE is calculated from a distribution of estimates so it also takes into account the precision of the method. Because the log-logistic model does not have a true threshold, two different approaches were used to estimate the RMSE: (i) assuming an apparent threshold equal to the threshold in the NEC models (i.e. 20%) in all curves, and (ii) assuming an apparent threshold equal to the true EC5 values of each log-logistic curve. Only the results from the apparent threshold equal to 20% are presented because there were no differences in the general trend of the results and, assuming an apparent threshold equal to the EC5 would inherently favor ECx analysis. The RMSE ratios among the designs were used to estimate the relative improvement in accuracy by each design and methods. Hence, RMSE ratios (RMSE_Categorical_/RMSE_Continuous_) should not deviate substantially from 1 if there is no difference in the accuracy of the design, and values higher than 1 would favor the denominator. The probability density distribution of the ECx and threshold estimates were plotted using Kernel density estimates with the beanplot R package [22]. High density intervals (HDI) were calculated for all thresholds and ECx estimates with the BEST R package [23]. Separate limits for discontinuous HDIs in multimodal distributions were not considered, so HDIs could be overestimated in these cases.

Model selection was used to identify the true and spurious incidences of threshold detection (i.e., detecting thresholds in datasets do not contain a true threshold). The AIC was calculated in the MLE NEC and models were compared to a three parameter log-logistic model. The DIC was calculated using MCMC in the Bayesian NEC and models were compared to a three parameter Bayesian log-logistic model. The piecewise regression was the only case where the two compared models had different numbers of parameters (i.e. with and without the threshold) so the AIC with a correction for finite sample sizes (AICc) was used. Note that model selection criteria using bias reduction methods is controversial [20]. However, very similar results were obtained with and without the bias reduction GLM fit and with the Konishi's generalized information criterion (GIC). Information criteria weights, which provides a relative weight of evidence for each model, was also used to estimate how many datasets had strong evidence (information criteria weights equal to or higher than 0.9) in support of a specific model [24]. All simulations and analysis were conducted using the R statistical environment software version 3.2.1[25].

## Results

### ECx analysis

**Fig 3:**
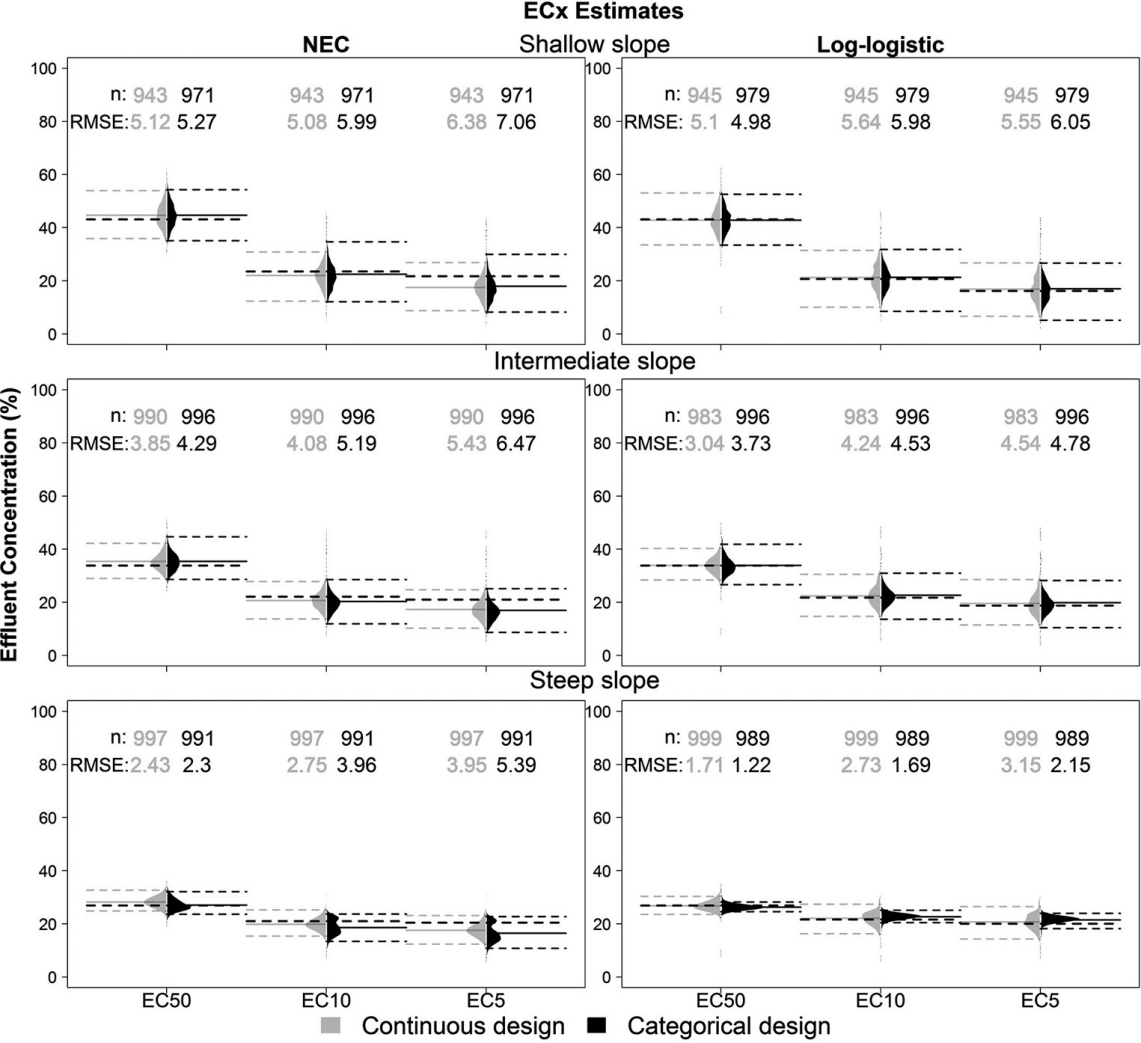
Distributions of the ECx estimates for the continuous and categorical designs (with medium background mortality) for three different types of curves (rows) and for the datasets generated from NEC and log-logistic models (columns). All models were fit with a three parameter log-logistic model. Black dashed lines indicate the true values of the ECx, and gray dashed lines indicate the 95% HDI. The RMSE and number of datasets are presented for each design.

When log-logistic models were fitted to the datasets with a threshold (i.e., NEC datasets), the EC50 was slightly overestimated and the EC5 and EC10 were underestimated (Fig 3). The distribution of EC5 and EC10 in the NEC datasets in the categorical design also tended to be bimodal for the steeply sloped curve. Due to the low number of concentrations used in the categorical design, stochastic variation may have a drastic impact in the curve fitting process of datasets with a threshold. The ECx estimates were generally more accurate in the datasets generated with the log-logistic models, especially in datasets with a steep slope. The EC50 estimates were more precise and accurate estimates in comparison to the EC5 and EC10 for all slopes and background mortality. The number of datasets that fitted to the model, accuracy and precision also increased with the slope of the curves. The mean ECx estimates were similar in all levels of background mortality; however, the 95% high density intervals became wider with the increase in background mortality. Consequently, the RMSE estimates also increased (S1 and S2 Figs in S2 Appendix).

In most cases, the RMSE ratio did not deviate substantially from 1 but favored the continuous design most of the time (S1 Table in S3 Appendix). The RMSE of the EC5 and EC10 estimates of the continuous design in datasets with a steep slope were, on average, 1.3 and 1.37 higher in the datasets with a threshold parameter. The RMSE ratios also increased with the slope. However, the categorical design was more accurate, with lower RMSE estimates by factors ranging from 0.62 to 0.84, in the ECx estimates in the log-logistic datasets with a steep slope (S1 Table in S3 Appendix). This was the only instance where the categorical design outperformed the continuous design in the ECx analysis. The mean estimates of the slope were generally overestimated in log-logistic datasets and underestimated in the NEC datasets (S4 Appendix).

### Threshold analysis

**Fig 4:**
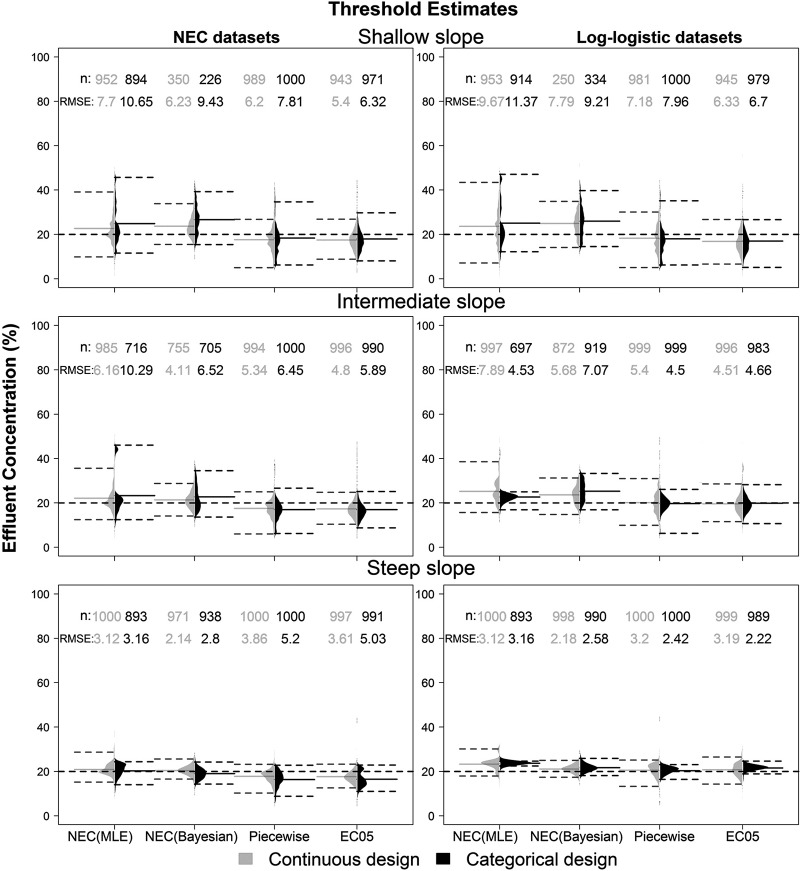
Distribution of the threshold and EC5 estimates for the continuous and categorical designs (with medium background mortality) for three different types of curves and for the datasets generated from NEC and log-logistic curves. Dashed lines represent the true threshold value for the NEC datasets and the apparent threshold in log-logistic datasets, assuming an apparent threshold equal to the NEC models. The numbers above the boxplots are the RMSE of the estimates and the number datasets that the method fitted to the data.

Both accuracy and precision of all threshold estimates also increased with the slope of the curves (Fig 4). All methods evaluated in this study had very poor performance (i.e. high RMSE and HDIs) in datasets with shallow slopes. Increases in the background mortality also led to an increase in the 95% HDIs and RMSE estimates (S1 and S2 Figs in S5 Appendix).

The piecewise regression was the only method where the accuracy increased with background mortality (Fig 4 and S1 and S2 Figs in S5 Appendix). In all scenarios, the piecewise regression underestimated the true threshold. The Bayesian estimation of the NEC model in datasets with a steep slope was the most accurate of the methods considered in this paper. The number of models that the Bayesian NEC fit acceptably to the data decreased with the slope and with the increase in background mortality. The estimates of the slope parameter were usually overestimated with the MLE NEC, especially for datasets generated with the log-logistic model. Higher estimates of the slope were also found in the datasets generated with log-logistic models with the Bayesian approach. Therefore, misspecification of the appropriate model resulted in a biased estimation of the slope parameter.

**Fig 5:**
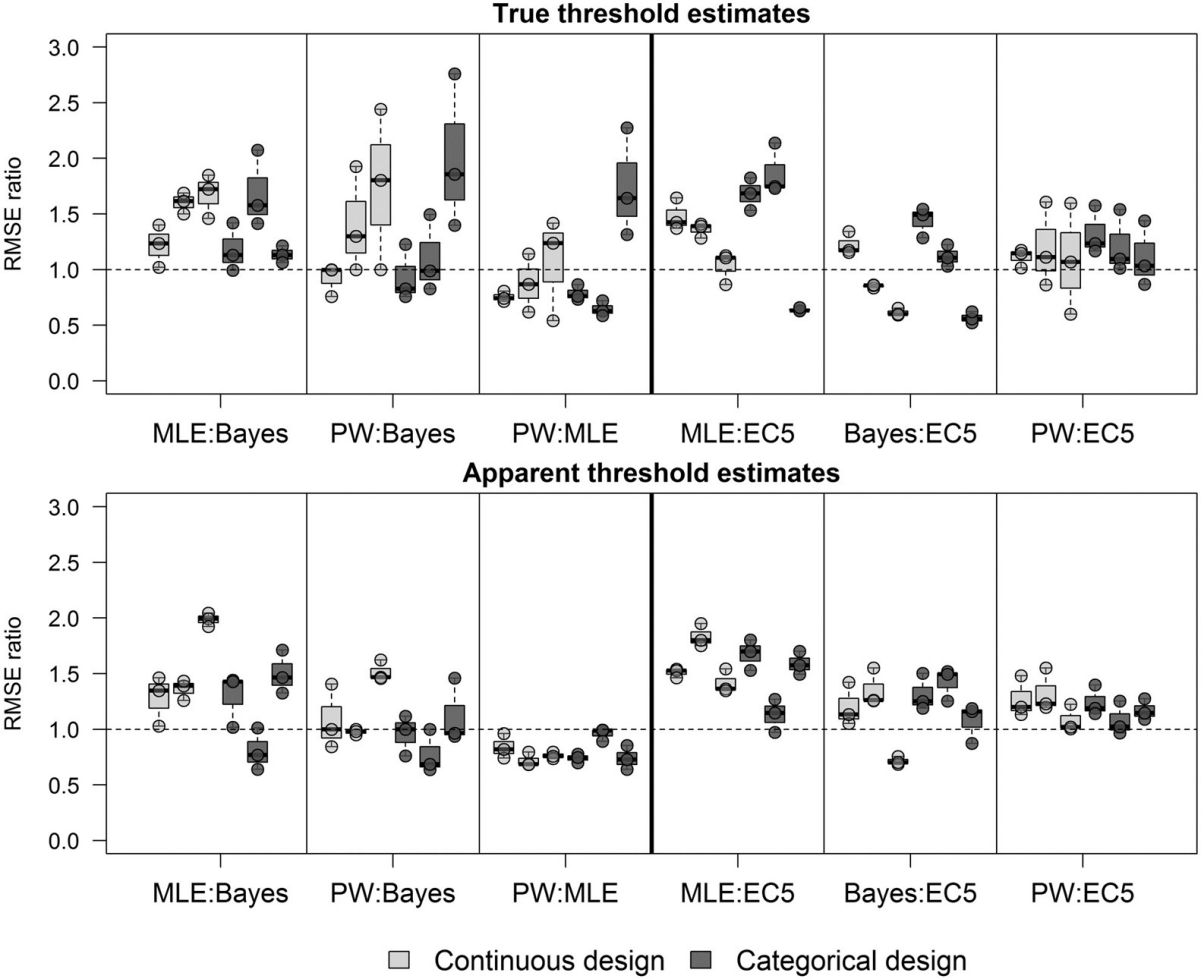
RMSE ratios between the MLE NEC, Bayesian NEC and EC5 estimates. Each point in the graphic represents one background mortality value (not differentiated in the figure) and each boxplot represents the shallow, intermediate and steep slopes (from the left to the right). Values above 1 (dashed line) favors the denominator.

In most cases, EC5 estimates from datasets with shallow and intermediate slopes were the most accurate and precise estimations of both true and apparent thresholds. However, the Bayesian and MLE NEC estimates outperformed the EC5 in datasets with a steep slope and a true threshold parameter (Fig 5). In this case, the MLE NEC in the continuous design with a steep slope had similar accuracy to the EC5, with the mean RMSE ratio close to 1. The piecewise regression only outperformed the EC5 in datasets with highest background mortality and a true threshold. The Bayesian NEC in the continuous design and steep slope was the only case where an apparent threshold was more accurate than the EC5. In datasets with a true threshold and a steep slope, the Bayesian NEC had on average RMSE values 1.67 times lower in comparison to the MLE NEC. In the categorical design, both MLE and Bayesian NEC had similar RMSE, being on average 1.64 times lower than the EC5.

**Fig 6:**
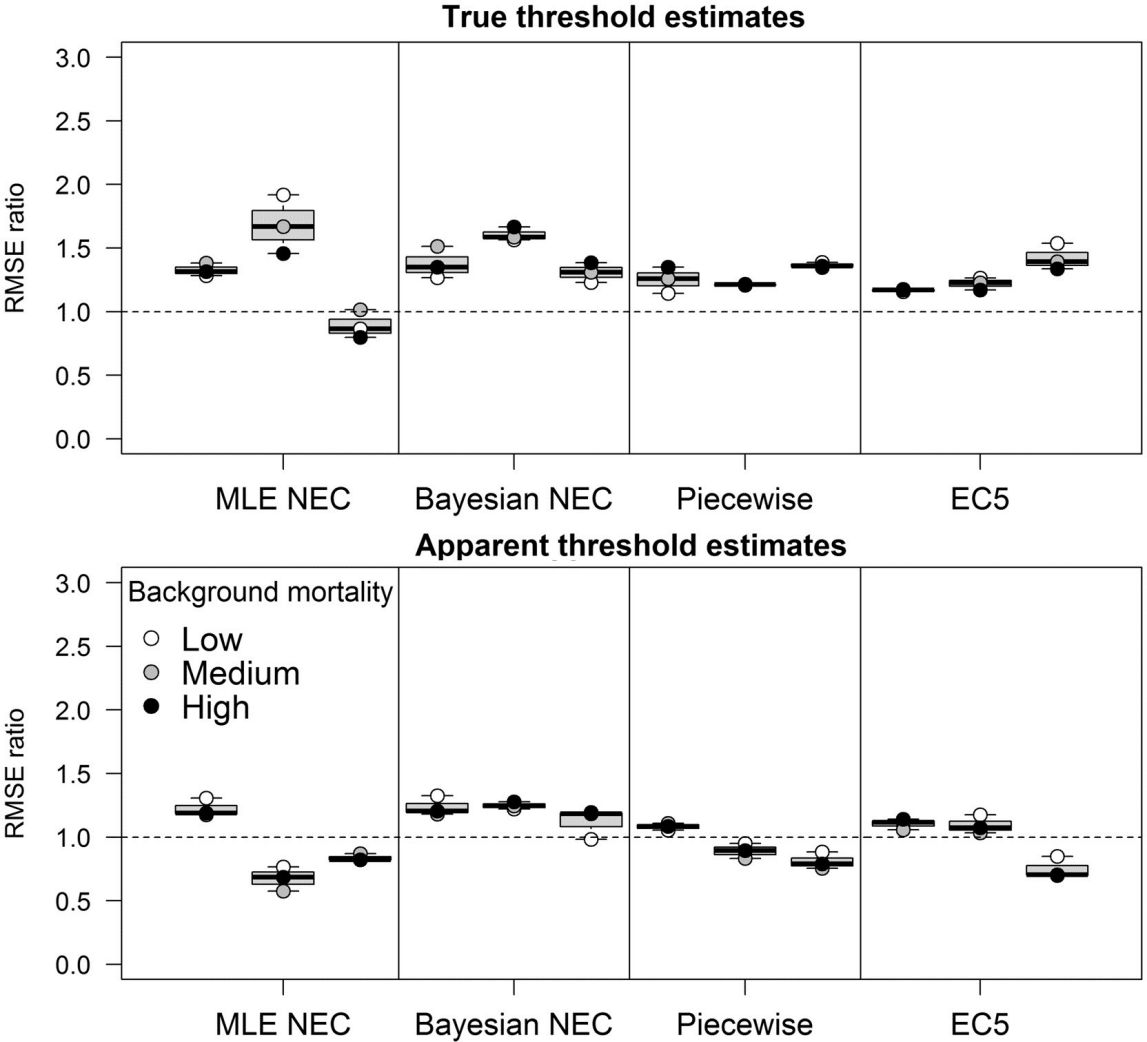
RMSE ratios between the categorical (numerator) and continuous designs (denominator) for the MLE NEC, Bayesian NEC, Piecewise regression and EC5. Each point in the graphic represents one background mortality, and each boxplot represents the shallow, intermediate and steep slopes (from the left to the right). Values above 1 (dashed line) favors the continuous design.

Regarding the threshold estimates among the two different designs, the RMSE of the continuous design was on average 1.31 lower than the RMSE of the categorical design with the Bayesian method in datasets with a true threshold parameter and a steep slope (Fig 6). In the log-logistic datasets, there was not a strong support for the continuous design with an average RMSE ratio of 1.12. In the MLE NEC approach, the RMSEs of the categorical design were lower than the continuous design in the steep slope in both datasets. The piecewise regression was more accurate in the continuous design in datasets with a true threshold. However, for datasets without a true threshold, the categorical design the RMSEs became lower with the increase of the slope.

### Model selection

**Fig 7:**
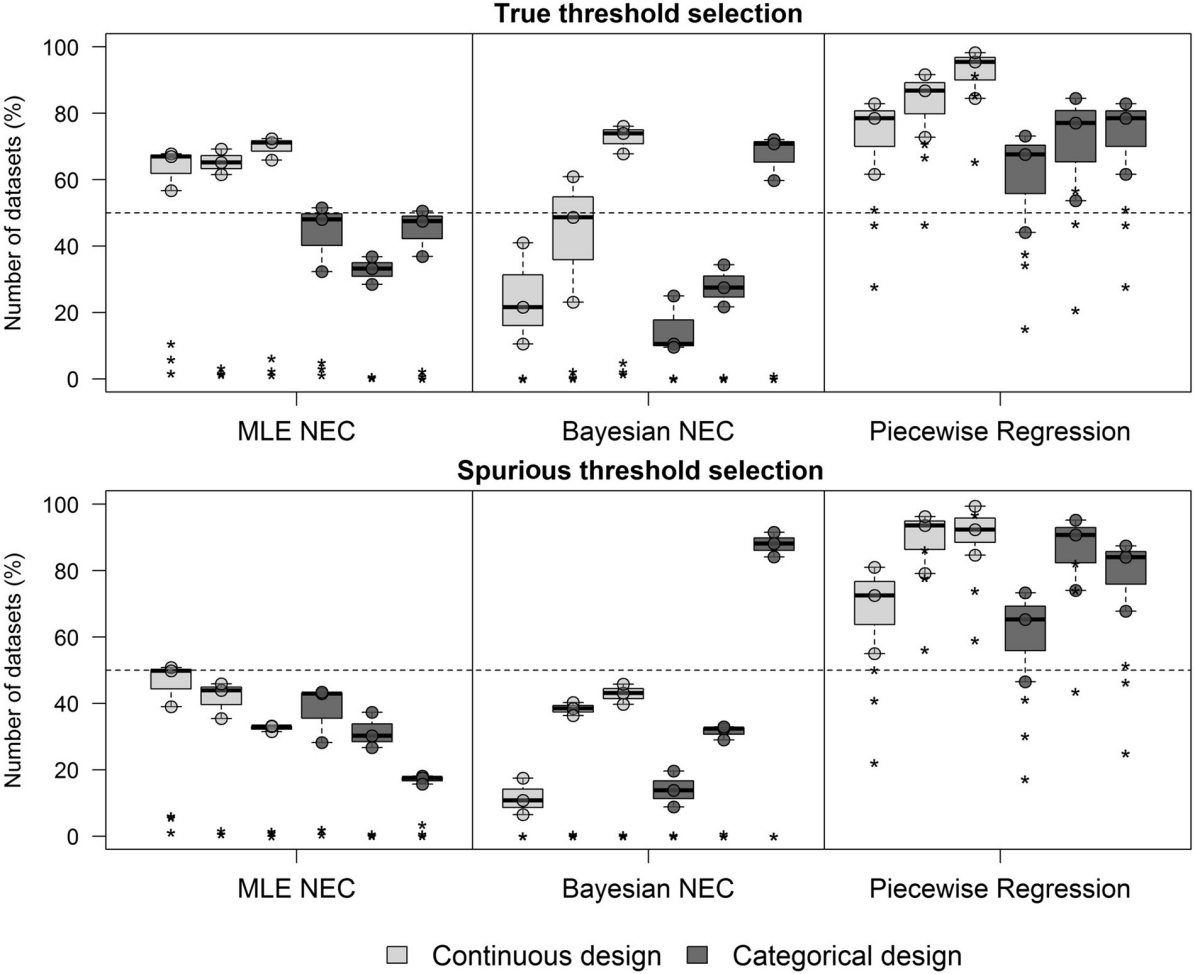
Rate of true and spurious threshold models selection from datasets with and without thresholds respectively. Each point in the graphic represents one background mortality value (not differentiated in the figure) and each boxplot represents the shallow, intermediate and steep slopes (from the left to the right). Asterisk marks represents the rate of model selection with the AIC, DIC and AICc weights for the MLE NEC, Bayesian NEC and Piecewise regression respectively. The dashed horizontal line is a reference line set at 50% of the datasets.

The rate of true threshold models selection with the Bayesian and Piecewise regression increased with the slope of the curve (Fig 7). The slope of the curve had a smaller effect on the model selection of the MLE NEC approach. In the MLE NEC, the threshold model was selected on average 66.3% of the datasets with the continuous design and 40.6% with the categorical design in all slopes. The continuous design usually had higher rates of true model selection in comparison to the categorical design. In the Bayesian NEC, the mean rates in datasets with a steep slope were 72.6% and 67.5% for the continuous and categorical design respectively. In the Piecewise regression, the rates were on average 92.7% and 74.3% in the continuous and categorical datasets with a steep slope. The increase in the background mortality generally decreased the rate of true threshold model selection, except for the piecewise regression where the rate increased with background mortality (S1, S2 and S3 Tables in S6 Appendix).

On the other hand, the rate of model selection with the AIC and DIC weights were much lower. In the MLE NEC approach, AIC weight was usually below 5% of the datasets with a mean of 6.1%, 2.1% and 3.2% in the shallow, intermediate, and steep curve of the continuous design, respectively. In the categorical design, this rate was even lower with 1.1%, 0.4%, and 1.1% in the shallow, intermediate and steep curve, respectively. In the Bayesian approach, most DIC weights were below 1% in both designs. The piecewise regression had a higher model selection rate with the AICc weights in datasets with a steep slope with a mean of 80.4% and 41.7% in the continuous and categorical design respectively. Usually, the AIC and DIC weights provided higher support for the log-logistic models in shallow and intermediate slopes and were higher than the rate of support to the NEC models in all cases (S1, S2 and S3 Tables in S6 Appendix).

The rates of spurious threshold model selection also increased with the slope of the curve in the Bayesian NEC and piecewise regression but decreased in the MLE NEC approach (Fig 7). The rates of spurious threshold detection were generally also higher with the continuous design, except for datasets with a steep slope in the Bayesian NEC approach, which had a mean of 87.9% in the categorical and 42.9% in the continuous. The rates of spurious model selection with the piecewise regression were even higher than the rates of true threshold selection in datasets with a steep slope, with a mean of 79.7% and 92.1% in the categorical and continuous design respectively. The AICc weights also had much higher values in comparison to the Bayesian and MLE NEC approach, with a mean of 40.9% and 75.6% in datasets with a steep slope in the categorical and continuous design respectively. The rates of spurious threshold model selection with the DIC weights were below 1% in all scenarios and designs. In the MLE NEC methods, the rates of spurious threshold model selection with the AIC weights was also low and below 2% in almost all scenarios. The DIC and AIC weights also supported log-logistic models more frequently than NEC models.

## Discussion

Threshold estimates in ecotoxicology have been proposed as an alternative to ECx and NOEC estimates. Different methods have been used to estimate these thresholds, such as the MLE NEC [9], Bayesian NEC [10], and piecewise regression [11]. The application of other analysis such as the binomial cumulative sum control chart (CUSUM) analysis [26], receiver operating characteristic (ROC) curves [27], and changepoint analysis [28] were also evaluated in earlier versions of this work. Even though they might be useful in detecting break points in ecotoxicology, their application in analyzing survival data is limited. These methods are mathematically and conceptually different from the previous described methods and thus not included in this work. In this paper, the accuracies of three different methods were compared to each other and to EC5 estimates under different scenarios and designs. Overall, the three methods were less accurate than the EC5 estimates in datasets with a shallow and intermediate curve, even in datasets which contained true thresholds. Based on these results, there seems to be no advantage in using any of these methods instead of ECx analysis in datasets with shallow and intermediate slopes.

A previous simulation study showed that piecewise regressions can provide accurate estimates of thresholds [29]. In the present work, piecewise regressions were usually less accurate than log logistic EC5 estimates and underestimated the true thresholds. However, estimates of the apparent threshold with the piecewise regressions were more accurate than the MLE NEC and, in some cases, the Bayesian NEC. One disadvantage of the piecewise regression is that threshold estimates were more affected by the effects of background mortality than are the other methods. Overall, increases in the background mortality lead to an increase in the HDI of all methods, which is in agreement with Bass et al. [7] who reported that increases in control mortality can make the estimation of the NEC more difficult. Besides, background mortality also affected the rates of true and spurious threshold models selection in all evaluated methods.

Of all the methods evaluated in this paper, the Bayesian NEC was the most accurate method in datasets with a steep slope. The Bayesian approach also has a series of advantages such as the direct inclusion of uncertainty in the estimates of the threshold parameter, which can be draw from the posterior distribution. Another advantage is that prior information can also be adjusted by using expert elicitation (e.g., [8]), information from the literature, or previous experiments. For instance, information about background mortality can be easily gathered for commonly used species in ecotoxicology. Even though weakly informative priors for all parameters were used in this work, it is likely that the inclusion of priors in the model fitting process contributed to the accuracy of the method. For instance, assuming flat priors with predefined upper and lower boundaries for the slope may have contributed to the overall model accuracy. One disadvantage of the Bayesian NEC is that the number of models that fit the data acceptably decreased with the slope, especially with higher background mortality.

Regarding the true threshold estimates, the only scenarios where threshold methods were more accurate than the EC5 were with the Bayesian and MLE NEC in datasets with a steep slope and a true threshold, and with the piecewise regression with high background mortality. Nevertheless, in such cases, the EC5 underestimated the true threshold, with a mean value of approximately 17% in both designs and background mortalities. In most cases, the widths of the HDIs of the Bayesian, MLE and EC5 within the same design were in the same range (Fig 4 and S4 Appendix). This indicates that the main driver of the lower RMSE values of the EC5 in datasets with a true threshold and a steep slope is the underestimation of the threshold and not the lack of precision of the method. Thus, if EC5 analyses are used in datasets with a true threshold, the EC5 is expected to on average, underestimate the threshold value. This is a reasonable result when the shape of both the log-logistic curve and the NEC curves are compared (Fig 1 and Table 1).

The Bayesian NEC in the continuous design with a steep slope was also the only method where apparent threshold estimates were more accurate than EC5 estimates. In this case, both methods had very close mean estimates of the apparent threshold with 20.78% and 21.08% respectively. However, the HDIs estimates were wider for the EC5 in comparison to the Bayesian approach. This was not observed in the categorical design which EC5 estimates had very precise and accurate estimates. Thus, from a practical point of view, there seems to be no advantage in estimating an apparent threshold instead of an EC5 in almost all scenarios evaluated in this work. In fact, the MLE NEC overestimated the apparent threshold in steep slopes with a mean of around 23% of the effluent in both designs. Overestimation and underestimation of the threshold value in relation to the EC5 may occur and were also observed by Forfait-Dubuc [8] with real datasets.

The rate of spurious threshold model selection based solely on the information criteria might be as high as 99.4% or as low as 6.5% depending on the method, slope, and design (Fig 7). The piecewise regression had the highest rates of spurious threshold detection and was the only method for which the information criteria weights provided high support for the threshold model. Daily et al [30] also reported high rates of spurious threshold detection in multivariate simulated datasets with piecewise quantile regression. The information criteria weights with the Bayesian and MLE NEC had rates of true and false threshold model selection usually below 5% and, in most cases, below 1%. Hence, there seems to be weak evidence in favor of one model over the other as pointed out by Ulm [12]. This is especially true in datasets with steep slopes, which had the lowest information criteria weights and in cases where the threshold is overestimated. As discussed by Fox [10], the introduction of a threshold parameter in the model does not presuppose the existence of a threshold, but just allows it to be estimated. However, the identification of the correct model is a hard task.

Regarding the experimental designs, the RMSEs ratios favored the continuous designs in most cases, but not in all cases. For instance, in the steep slope, the categorical design was more accurate than the continuous design with the MLE NEC method, and the opposite occurred with the Bayesian NEC. The main idea of favoring the number of concentrations instead of the number of replicates per treatment is that it might increase the accuracy in estimating the shape of the curve [31] which can increase the ability to estimating thresholds [11]. One example of the problem with fitting threshold model with low number of concentrations and a shallow slope can be illustrated in Fig 2A, where the log-logistic and NEC models provided very different results. In this example, the NEC model would predict a much higher threshold (39.1% of the effluent) and the log logistic model is only weakly favored by the AIC weights (i.e. 0.54). In such cases, where the shape of the curve is not clear, more data should be gathered if threshold models are going to be used.

The low number of sampling replicates (i.e., organisms) per concentration in the continuous design might also make it difficult to precisely estimate the threshold or ECx value, especially if there is high background mortality. Thus, experimental designs should balance the number of concentrations and sampling replicates in a way that it maximizes the accuracy of the statistical method. Because there are innumerable design permutations and design will also depend on the funding of the study, pilot and simulation studies are recommended if threshold models are going to be used. Also, dose response curves may present a wide range of slopes depending on the test organisms, contaminant of interest and their modes of action [32]. Because organisms may present different sensitivity to contaminants (i.e., ranging from μg/L to mg/L), the interpretation of steepness of the slope may be ambiguous when comparing curves with different concentration units. A steep slope, in the context of this work, should be interpreted based on the shape of the dose response curve, independent of the concentration unit or x-axis scale. Future studies should also evaluate the accuracy of ECx estimates in relation to (i) different threshold models (such as the time-independent NEC and models that assume triangular distributions), and (ii) other distributions, such as Gaussian and Poisson.

## Conclusion

Thresholds are an attractive concept from a regulatory and practical point of view. However, threshold estimates might not be reasonable in all scenarios, such as when the data have shallow or intermediate slopes. In most scenarios, EC5 estimates were the most accurate method. Nevertheless, EC5 may underestimate the true threshold in steep slopes and in such scenario the Bayesian NEC was the most accurate methods. However, there seems to be no strong evidence in favor of either log-logistic or NEC models in all cases. Thus, selecting the correct models is an extremely hard task. The piecewise regression was the only method where the information criteria weights had higher support for the threshold model; however, the rates of spurious threshold selection were also high. Measuring an apparent threshold does not seem to have any advantage over the EC5 in most cases, and in fact, it can overestimate the apparent threshold. Hence, threshold models should be used carefully or when there are any biological reasons to support the existence of a threshold. In such cases, more data should be gathered around the estimated threshold to better understand the shape of the dose-response curve and the mechanisms behind threshold effects.

## Supporting information

S1 Appendix (DOCX)

S2 Appendix (DOCX)

S3 Appendix (DOCX)

S4 Appendix (DOCX)

S5 Appendix (DOCX)

S6 Appendix (DOCX)

## References

[1] Finney DJ. Probit analysis: a statistical treatment of the sigmoid response curve. 1st ed New York: Cambridge University Press; 1947.

[2] Rall DP. Thresholds? Environ Health Perspect. 1978; 22: 63–165. 10.1289/ehp.7822163 PMC1637159 648484

[3] Kooijman SALM. Parametric analyses of mortality rates in bioassays. Wat Res 1981; 15: 107–119.

[4] Cox C. Threshold Dose-response models in toxicology. Biometrics. 1987; 43: 511–523. 3663815

[5] Fox DR. NECS, NOECS and ECx. Australas J Ecotoxicol. 2008; 14: 7–9.

[6] Kooijman SALM, Bedaux JJM, Slob W. No-Effect concentration as a basis for ecological risk assessment. Risk Analysis. 1996; 16: 445–447. 10.1111/j.1539-6924.1996.tb01091.x 8819337

[7] Baas J, Jager T, Kooijman SALM. Estimation of no effect concentrations from exposure experiments when values scatter among individuals. Ecol model. 2009; 220: 411–418.

[8] Forfait-Dubuc C, Charles S, Billoir E, Delignette-Muller ML. Survival data analyses in ecotoxicology: critical effect concentrations, methods and models. What should we use? Ecotoxicology. 2012; 21: 1972–1083. 10.1007/s10646-012-0860-0 22302371

[9] Pires AM, Branco JA, Picado A, Mendonça E. Models for the estimation of a ‘no effect concentration’. Environmetrics. 2002; 13: 15–27.

[10] Fox DR. A Bayesian approach for determining the no effect concentration and hazardous concentration in ecotoxicology. Ecotoxicol Environ Saf. 2010; 73: 123–131. 10.1016/j.ecoenv.2009.09.012 19836077

[11] Mebane CA. In Response: Biological arguments for selecting effect sizes in ecotoxicological testing—A governmental perspective. Environ Toxicol Chem. 2015; 34: 2440–2442. 10.1002/etc.3108 26496135

[12] Ulm K. “Comment on”: On the estimation of threshold values. Biometrics. 1989; 45: 1324–1326.

[13] Cox C. Response to “Comment on”: On the estimation of threshold values. Biometrics. 1989; 45: 1327–1328.

[14] Ritz C. Toward a unified approach to dose-response modeling in ecotoxicology. Environ Toxicol Chem. 2010; 29: 220−229. 10.1002/etc.7 20821438

[15] Weber CI. Methods for measuring the acute toxicity of effluents and receiving waters to freshwater and marine organisms. EPA 821-R-02-012, U.S. Environmental Protection Agency; 2002.

[16] Ritz C, Streibig JC. Bioassay Analysis using R. J Statist Software. 2005; 12:1–22.

[17] Plummer M. rjags: Bayesian Graphical Models using MCMC. R package version 4–5. 2016; http://CRAN.R-project.org/package=rjags

[18] Firth D. Bias reduction of maximum likelihood estimates. Biometrika. 1993; 80: 27–38

[19] Heinze G, Schemper M. A solution to the problem of separation in logistic regression. Stat Med. 2002; 21: 2409–2419. 10.1002/sim.1047 12210625

[20] Kosmidis I. brglm: Bias reduction in binomial-response Generalized Linear Models. 2013; http://www.ucl.ac.uk/~ucakiko/software.html

[21] Muggeo VMR. Segmented: an R Package to Fit Regression Models with Broken-Line Relationships. R News. 2008; 8: 20–25. http://cran.r-project.org/doc/Rnews/

[22] Kampstra P. Beanplot: A Boxplot Alternative for Visual Comparison of Distributions. J Stat Softw. 2008; 28: 1–9. 10.18637/jss.v028.i07 27774042 PMC5074077

[23] Kruschke JK, Meredith M. BEST: Bayesian Estimation Supersedes the t-Test. R package version 0.4.0. 2015; http://CRAN.R-project.org/package=BEST

[24] Johnson JB, Omland KS. Model selection in ecology and evolution. Trends Ecol Evol. 2004; 19: 101–108. 10.1016/j.tree.2003.10.013 16701236

[25] R Core Team. R: A language and environment for statistical computing. R Foundation for Statistical Computing, Vienna, Austria 2016; URL https://www.R-project.org/

[26] Reynolds MR, Stoumbos ZG. A general approach to modeling CUSUM charts for a proportion. LIE Trans. 2000; 32: 515–535.

[27] Robin X, Turck N, Hainard A, Tiberti N, Lisacek F, Sanchez J, et al pROC: an open-source package for R and S+ to analyze and compare ROC curves. BMC Bioinformatics. 2011; 12: 1–8. 10.1186/1471-2105-12-1 21414208 PMC3068975

[28] Killick R, Eckley I. changepoint: An R package for changepoint analysis. J Stat Softw. 2014; 58: 1–19.

[29] Ficetola GF, Denoël M. Ecological thresholds: An assessment of methods to identify abrupt changes in species habitat relationships. Ecography. 2009; 32: 1075–1084.

[30] Daily JP, Hitt NP, Smith DR, Snyder CD. Experimental and environmental factors affect spurious detection of ecological thresholds. Ecology. 2012; 93: 17–23. 10.1890/11-0516.1 22486082

[31] Andersen JS, Holst H, Spliid H, Andersen H, Nyholm N, Baun A. Continuous ecotoxicological data evaluated relative to a control response. J Agri Biol Environ Stat. 1998; 3: 405–420.

[32] Smit MG, Hendriks AJ, Schobben JH, Karman CC, Schobben HP. The variation in slope of concentration–effect relationships. Ecotoxicol Environ Saf. 2001; 48: 43–50. 10.1006/eesa.2000.1983 11161676

