# Supplementary material for: Comparing statistical analyses to estimate thresholds in ecotoxicology
Source: PLoS One. 2020 Apr 8;15(4):e0231149. doi: 10.1371/journal.pone.0231149 (PMC7141675; doi:10.1371/journal.pone.0231149)
Supplement: S1 Appendix — (DOCX) [file pone.0231149.s001.docx]

**Appendix S1**

**Table S1.**

Table S1. Percent and standard deviations of the number of tests rejected based on the control survival of the three background mortality probabilities used in this work, assuming 90% and 80% of survival quality control (n=10^6^).

|  |  |  | Rejection rates | |
| --- | --- | --- | --- | --- |
| Background mortality | | | 90% of survival | 80% of survival |
| Low (0.95) | | | 6.12% (±0.74) | 0.06% (±0.07) |
| Medium (0.90) | | | 35.2% (±1.50) | 2.61% (±0.50) |
| High (0.85) | | | 67.8% (±1.46) | 15.2% (±1.12) |
