# Supplementary material for: Comparing statistical analyses to estimate thresholds in ecotoxicology
Source: PLoS One. 2020 Apr 8;15(4):e0231149. doi: 10.1371/journal.pone.0231149 (PMC7141675; doi:10.1371/journal.pone.0231149)
Supplement: S2 Appendix — (DOCX) [file pone.0231149.s002.docx]

**Appendix S2**

**Fig S1**

**
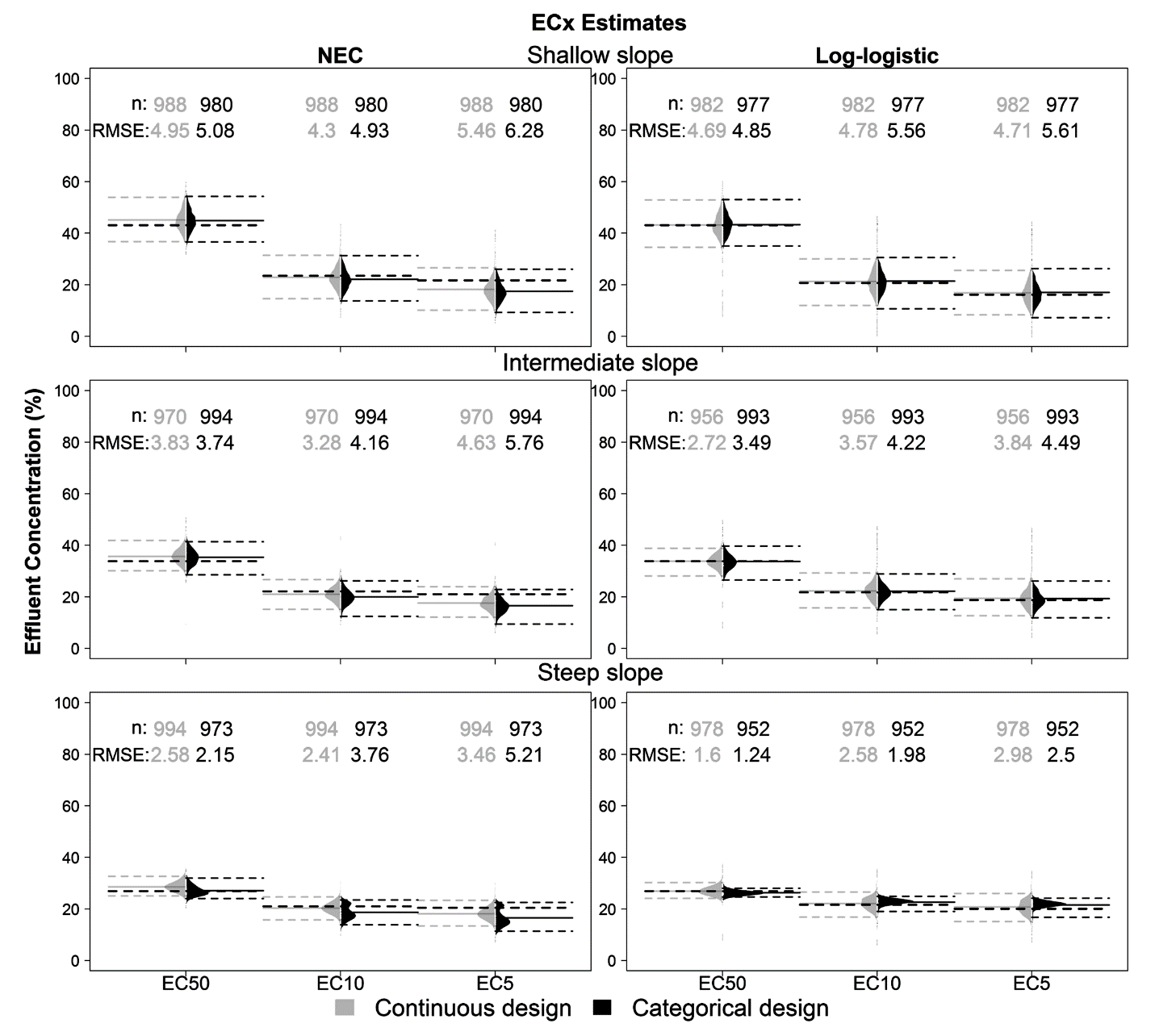
**

**Fig S1. Distributions of the ECx estimates for the continuous and categorical designs (with low background mortality) for three different types of curves and for the datasets generated from NEC and log-logistic models**. All models were fit with a three-parameter log-logistic model. Black dashed lines indicate the true values of the ECx, and gray dashed lines indicate the 95% HDI. The RMSE and number of datasets are presented for each design.

**Fig S2**

**
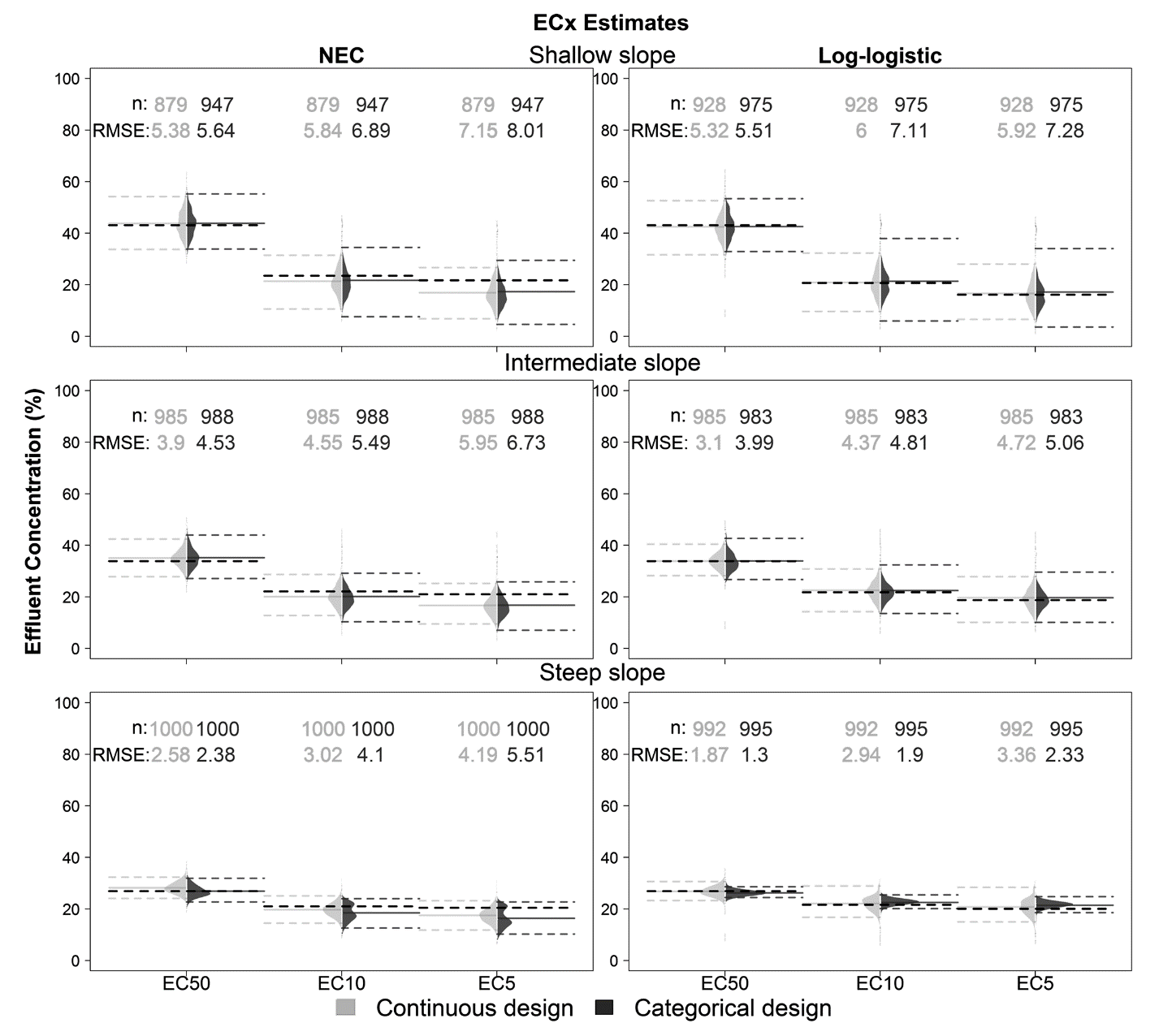
**

**Fig S2. Distributions of the ECx estimates for the continuous and categorical designs (with high background mortality) for three different types of curves and for the datasets generated from NEC and log-logistic models**. All models were fit with a three-parameter log-logistic model. Black dashed lines indicate the true values of the ECx, and gray dashed lines indicate the 95% HDI. The RMSE and number of datasets are presented for each design.
