# Supplementary material for: Comparing statistical analyses to estimate thresholds in ecotoxicology
Source: PLoS One. 2020 Apr 8;15(4):e0231149. doi: 10.1371/journal.pone.0231149 (PMC7141675; doi:10.1371/journal.pone.0231149)
Supplement: S3 Appendix — (DOCX) [file pone.0231149.s003.docx]

**Appendix S3**

**Table S1. Comparison of accuracy of the ECx estimates for the categorical and continuous design.** The mean RMSE all background mortalities were used for each type of datasets and curve. RMSE ratios higher than 1 indicate higher accuracy for the denominator.

|  |  | **Mean CV ratio (Categorical/Continuous)** | | | | | |
| --- | --- | --- | --- | --- | --- | --- | --- |
|  |  | **NEC** | | | **Logistic** | | |
| **Slope** | **Background mortality** | **EC50** | **EC10** | **EC5** | **EC50** | **EC10** | **EC5** |
| **Shallow** | **Low** | 1.06 | 1.12 | 1.11 | 1.03 | 1.16 | 1.19 |
|  | **Medium** | 1.03 | 1.21 | 1.26 | 0.98 | 1.06 | 1.09 |
|  | **High** | 1.05 | 1.22 | 1.26 | 1.04 | 1.18 | 1.22 |
|  | **Mean:** | **1.05** | **1.18** | **1.21** | **1.01** | **1.13** | **1.17** |
| **Intermediate** | **Low** | 1.03 | 1.18 | 1.18 | 1.29 | 1.19 | 1.18 |
|  | **Medium** | 1.13 | 1.28 | 1.27 | 1.22 | 1.06 | 1.04 |
|  | **High** | 1.17 | 1.27 | 1.28 | 1.28 | 1.10 | 1.07 |
|  | **Mean:** | **1.11** | **1.24** | **1.24** | **1.27** | **1.12** | **1.10** |
| **Steep** | **Low** | 1.15 | 1.34 | 1.43 | 0.68 | 0.68 | 0.70 |
|  | **Medium** | 1.17 | 1.33 | 1.39 | 0.64 | 0.50 | 0.51 |
|  | **High** | 1.11 | 1.24 | 1.30 | 0.61 | 0.58 | 0.58 |
|  | **Mean:** | **1.15** | **1.30** | **1.37** | **0.65** | **0.59** | **0.60** |
| **Ovearll Mean**  **[95% HDI]** | | 1.10  [1.1. 1.17] | 1.24  [1.11. 1.34] | 1.27  [1.11. 1.42] | 0.97  [0.61. 1.28] | 0.95  [0.5. 1.19] | 0.95  [0.51. 1.22] |
