# Supplementary material for: Comparing statistical analyses to estimate thresholds in ecotoxicology
Source: PLoS One. 2020 Apr 8;15(4):e0231149. doi: 10.1371/journal.pone.0231149 (PMC7141675; doi:10.1371/journal.pone.0231149)
Supplement: S4 Appendix — (DOCX) [file pone.0231149.s004.docx]

**Appendix S4**

**Table S1. Parameter estimates with a three-parameter log-logistic model for the shallow slope.** Type of data indicates if datasets are either log-logistic (LL) or contain a threshold (NEC).

| **Type of Curve** | **Type of Data** | **Design** | **Parameter** | **Background Mortality** | **True Value** | **Mean** | **HDI 95% lower** | **HDI 95% Upper** |
| --- | --- | --- | --- | --- | --- | --- | --- | --- |
| Shallow | LL | Cat | Slope | Low | 3 | 3.48 | 1.71 | 4.98 |
| Shallow | LL | Cat | Slope | Medium | 3 | 3.45 | 1.55 | 5.16 |
| Shallow | LL | Cat | Slope | High | 3 | 3.67 | 1.21 | 8.50 |
| Shallow | LL | Cat | Intercept | Low | 0.95 | 0.96 | 0.89 | 1.01 |
| Shallow | LL | Cat | Intercept | Medium | 0.90 | 0.91 | 0.83 | 0.97 |
| Shallow | LL | Cat | Intercept | High | 0.85 | 0.86 | 0.78 | 0.94 |
| Shallow | LL | Cat | EC50 | Low | 43.1 | 43.29 | 35.05 | 53.12 |
| Shallow | LL | Cat | EC50 | Medium | 43.1 | 42.84 | 33.42 | 52.58 |
| Shallow | LL | Cat | EC50 | High | 43.1 | 42.63 | 32.89 | 53.30 |
| Shallow | LL | Cont | Slope | Low | 3 | 3.22 | 1.68 | 5.07 |
| Shallow | LL | Cont | Slope | Medium | 3 | 3.31 | 1.45 | 5.37 |
| Shallow | LL | Cont | Slope | High | 3 | 3.31 | 1.35 | 5.52 |
| Shallow | LL | Cont | Intercept | Low | 0.95 | 0.95 | 0.90 | 1.00 |
| Shallow | LL | Cont | Intercept | Medium | 0.90 | 0.90 | 0.84 | 0.97 |
| Shallow | LL | Cont | Intercept | High | 0.85 | 0.86 | 0.78 | 0.93 |
| Shallow | LL | Cont | EC50 | Low | 43.1 | 43.14 | 33.75 | 52.53 |
| Shallow | LL | Cont | EC50 | Medium | 43.1 | 42.89 | 33.77 | 53.08 |
| Shallow | LL | Cont | EC50 | High | 43.1 | 42.56 | 31.93 | 52.83 |
| Shallow | NEC | Cat | Slope | Low | 3 | 3.39 | 1.91 | 4.79 |
| Shallow | NEC | Cat | Slope | Medium | 3 | 3.48 | 1.63 | 5.21 |
| Shallow | NEC | Cat | Slope | High | 3 | 3.48 | 1.38 | 5.33 |
| Shallow | NEC | Cat | Intercept | Low | 0.95 | 0.96 | 0.90 | 1.01 |
| Shallow | NEC | Cat | Intercept | Medium | 0.90 | 0.91 | 0.84 | 0.97 |
| Shallow | NEC | Cat | Intercept | High | 0.85 | 0.87 | 0.80 | 0.95 |
| Shallow | NEC | Cat | EC50 | Low | 43.1 | 44.91 | 36.62 | 54.35 |
| Shallow | NEC | Cat | EC50 | Medium | 43.1 | 44.70 | 35.11 | 54.24 |
| Shallow | NEC | Cat | EC50 | High | 43.1 | 43.90 | 33.84 | 55.21 |
| Shallow | NEC | Cont | Slope | Low | 3 | 3.34 | 1.78 | 4.68 |
| Shallow | NEC | Cont | Slope | Medium | 3 | 3.23 | 1.80 | 4.91 |
| Shallow | NEC | Cont | Slope | High | 3 | 3.21 | 1.66 | 5.14 |
| Shallow | NEC | Cont | Intercept | Low | 0.95 | 0.96 | 0.91 | 1.00 |
| Shallow | NEC | Cont | Intercept | Medium | 0.90 | 0.91 | 0.86 | 0.98 |
| Shallow | NEC | Cont | Intercept | High | 0.85 | 0.87 | 0.80 | 0.94 |
| Shallow | NEC | Cont | EC50 | Low | 43.1 | 45.11 | 36.30 | 53.79 |
| Shallow | NEC | Cont | EC50 | Medium | 43.1 | 44.70 | 36.00 | 53.97 |
| Shallow | NEC | Cont | EC50 | High | 43.1 | 43.83 | 33.78 | 54.24 |

**Table S2. Parameter estimates with a three parameter log-logistic model for the intermediate slope.** Type of data indicates if datasets are either log-logistic (LL) or contain a threshold (NEC).

| **Type of Curve** | **Type of Data** | **Design** | **Parameter** | **Background Mortality** | **True Value** | **Mean** | **HDI 95% lower** | **HDI 95% Upper** |
| --- | --- | --- | --- | --- | --- | --- | --- | --- |
| Interm | LL | Cat | Slope | Low | 5 | 6.86 | 2.89 | 19.38 |
| Interm | LL | Cat | Slope | Medium | 5 | 6.46 | 2.83 | 16.14 |
| Interm | LL | Cat | Slope | High | 5 | 6.23 | 2.77 | 15.81 |
| Interm | LL | Cat | Intercept | Low | 0.95 | 0.95 | 0.89 | 0.99 |
| Interm | LL | Cat | Intercept | Medium | 0.9 | 0.90 | 0.83 | 0.96 |
| Interm | LL | Cat | Intercept | High | 0.85 | 0.86 | 0.79 | 0.93 |
| Interm | LL | Cat | EC50 | Low | 33.86 | 34.23 | 26.64 | 44.83 |
| Interm | LL | Cat | EC50 | Medium | 33.86 | 34.17 | 26.78 | 43.25 |
| Interm | LL | Cat | EC50 | High | 33.86 | 34.08 | 26.30 | 42.81 |
| Interm | LL | Cont | Slope | Low | 5 | 5.86 | 3.08 | 9.91 |
| Interm | LL | Cont | Slope | Medium | 5 | 6.00 | 2.68 | 10.11 |
| Interm | LL | Cont | Slope | High | 5 | 6.32 | 2.61 | 10.39 |
| Interm | LL | Cont | Intercept | Low | 0.95 | 0.95 | 0.91 | 1.00 |
| Interm | LL | Cont | Intercept | Medium | 0.9 | 0.90 | 0.84 | 0.96 |
| Interm | LL | Cont | Intercept | High | 0.85 | 0.86 | 0.79 | 0.92 |
| Interm | LL | Cont | EC50 | Low | 33.86 | 33.88 | 27.98 | 38.89 |
| Interm | LL | Cont | EC50 | Medium | 33.86 | 33.91 | 28.45 | 40.23 |
| Interm | LL | Cont | EC50 | High | 33.86 | 33.87 | 28.26 | 40.40 |
| Interm | NEC | Cat | Slope | Low | 5 | 4.45 | 2.05 | 6.42 |
| Interm | NEC | Cat | Slope | Medium | 5 | 4.54 | 2.15 | 6.72 |
| Interm | NEC | Cat | Slope | High | 5 | 4.43 | 1.42 | 7.25 |
| Interm | NEC | Cat | Intercept | Low | 0.95 | 0.96 | 0.90 | 1.00 |
| Interm | NEC | Cat | Intercept | Medium | 0.9 | 0.91 | 0.84 | 0.97 |
| Interm | NEC | Cat | Intercept | High | 0.85 | 0.86 | 0.79 | 0.94 |
| Interm | NEC | Cat | EC50 | Low | 33.86 | 35.74 | 26.88 | 42.44 |
| Interm | NEC | Cat | EC50 | Medium | 33.86 | 35.60 | 27.94 | 44.80 |
| Interm | NEC | Cat | EC50 | High | 33.86 | 35.41 | 27.74 | 45.92 |
| Interm | NEC | Cont | Slope | Low | 5 | 4.30 | 2.56 | 6.25 |
| Interm | NEC | Cont | Slope | Medium | 5 | 4.45 | 2.23 | 6.72 |
| Interm | NEC | Cont | Slope | High | 5 | 4.12 | 2.28 | 6.69 |
| Interm | NEC | Cont | Intercept | Low | 0.95 | 0.97 | 0.91 | 1.00 |
| Interm | NEC | Cont | Intercept | Medium | 0.9 | 0.91 | 0.86 | 0.97 |
| Interm | NEC | Cont | Intercept | High | 0.85 | 0.87 | 0.80 | 0.94 |
| Interm | NEC | Cont | EC50 | Low | 33.86 | 35.72 | 30.06 | 41.95 |
| Interm | NEC | Cont | EC50 | Medium | 33.86 | 35.45 | 28.99 | 42.24 |
| Interm | NEC | Cont | EC50 | High | 33.86 | 35.11 | 27.72 | 42.13 |

**Table S3. Parameter estimates with a three parameter log-logistic model for the steep slope.** Type of data indicates if datasets are either log-logistic (LL) or contain a threshold (NEC).

| **Type of Curve** | **Type of Data** | **Design** | **Parameter** | **Background Mortality** | **True Value** | **Mean** | **HDI 95% lower** | **HDI 95% Upper** |  |
| --- | --- | --- | --- | --- | --- | --- | --- | --- | --- |
| Steep | LL | Cat | Slope | Low | 10 | 18.31 | 3.93 | 39.91 | |
| Steep | LL | Cat | Slope | Medium | 10 | 15.97 | 5.50 | 19.22 | |
| Steep | LL | Cat | Slope | High | 10 | 15.51 | 5.35 | 18.88 | |
| Steep | LL | Cat | Intercept | Low | 0.95 | 0.95 | 0.88 | 0.99 | |
| Steep | LL | Cat | Intercept | Medium | 0.9 | 0.90 | 0.84 | 0.96 | |
| Steep | LL | Cat | Intercept | High | 0.85 | 0.86 | 0.79 | 0.92 | |
| Steep | LL | Cat | EC50 | Low | 26.93 | 26.69 | 24.61 | 30.71 | |
| Steep | LL | Cat | EC50 | Medium | 26.93 | 26.43 | 24.58 | 28.62 | |
| Steep | LL | Cat | EC50 | High | 26.93 | 26.28 | 24.47 | 28.63 | |
| Steep | LL | Cont | Slope | Low | 10 | 15.89 | 5.16 | 46.41 | |
| Steep | LL | Cont | Slope | Medium | 10 | 16.09 | 5.13 | 42.98 | |
| Steep | LL | Cont | Slope | High | 10 | 15.97 | 4.00 | 42.49 | |
| Steep | LL | Cont | Intercept | Low | 0.95 | 0.95 | 0.91 | 0.99 | |
| Steep | LL | Cont | Intercept | Medium | 0.9 | 0.90 | 0.84 | 0.96 | |
| Steep | LL | Cont | Intercept | High | 0.85 | 0.86 | 0.79 | 0.92 | |
| Steep | LL | Cont | EC50 | Low | 26.93 | 27.03 | 24.07 | 30.32 | |
| Steep | LL | Cont | EC50 | Medium | 26.93 | 26.78 | 23.57 | 30.32 | |
| Steep | LL | Cont | EC50 | High | 26.93 | 26.81 | 23.27 | 30.60 | |
| Steep | NEC | Cat | Slope | Low | 10 | 8.46 | 2.80 | 18.24 | |
| Steep | NEC | Cat | Slope | Medium | 10 | 7.76 | 3.17 | 17.36 | |
| Steep | NEC | Cat | Slope | High | 10 | 7.64 | 3.13 | 16.97 | |
| Steep | NEC | Cat | Intercept | Low | 0.95 | 0.96 | 0.90 | 1.01 | |
| Steep | NEC | Cat | Intercept | Medium | 0.9 | 0.91 | 0.84 | 0.97 | |
| Steep | NEC | Cat | Intercept | High | 0.85 | 0.86 | 0.80 | 0.94 | |
| Steep | NEC | Cat | EC50 | Low | 26.93 | 27.40 | 23.32 | 32.09 | |
| Steep | NEC | Cat | EC50 | Medium | 26.93 | 27.21 | 23.29 | 32.08 | |
| Steep | NEC | Cat | EC50 | High | 26.93 | 26.95 | 22.77 | 31.96 | |
| Steep | NEC | Cont | Slope | Low | 10 | 7.20 | 3.66 | 11.52 | |
| Steep | NEC | Cont | Slope | Medium | 10 | 7.03 | 3.45 | 12.02 | |
| Steep | NEC | Cont | Slope | High | 10 | 6.81 | 3.44 | 11.22 | |
| Steep | NEC | Cont | Intercept | Low | 0.95 | 0.96 | 0.91 | 0.99 | |
| Steep | NEC | Cont | Intercept | Medium | 0.9 | 0.91 | 0.86 | 0.96 | |
| Steep | NEC | Cont | Intercept | High | 0.85 | 0.86 | 0.80 | 0.93 | |
| Steep | NEC | Cont | EC50 | Low | 26.93 | 28.60 | 25.05 | 32.79 | |
| Steep | NEC | Cont | EC50 | Medium | 26.93 | 28.28 | 24.77 | 32.63 | |
| Steep | NEC | Cont | EC50 | High | 26.93 | 28.22 | 24.17 | 32.40 | |
