# Supplementary material for: Comparing statistical analyses to estimate thresholds in ecotoxicology
Source: PLoS One. 2020 Apr 8;15(4):e0231149. doi: 10.1371/journal.pone.0231149 (PMC7141675; doi:10.1371/journal.pone.0231149)
Supplement: S5 Appendix — (DOCX) [file pone.0231149.s005.docx]

**Appendix S5**

**Fig S1**

**
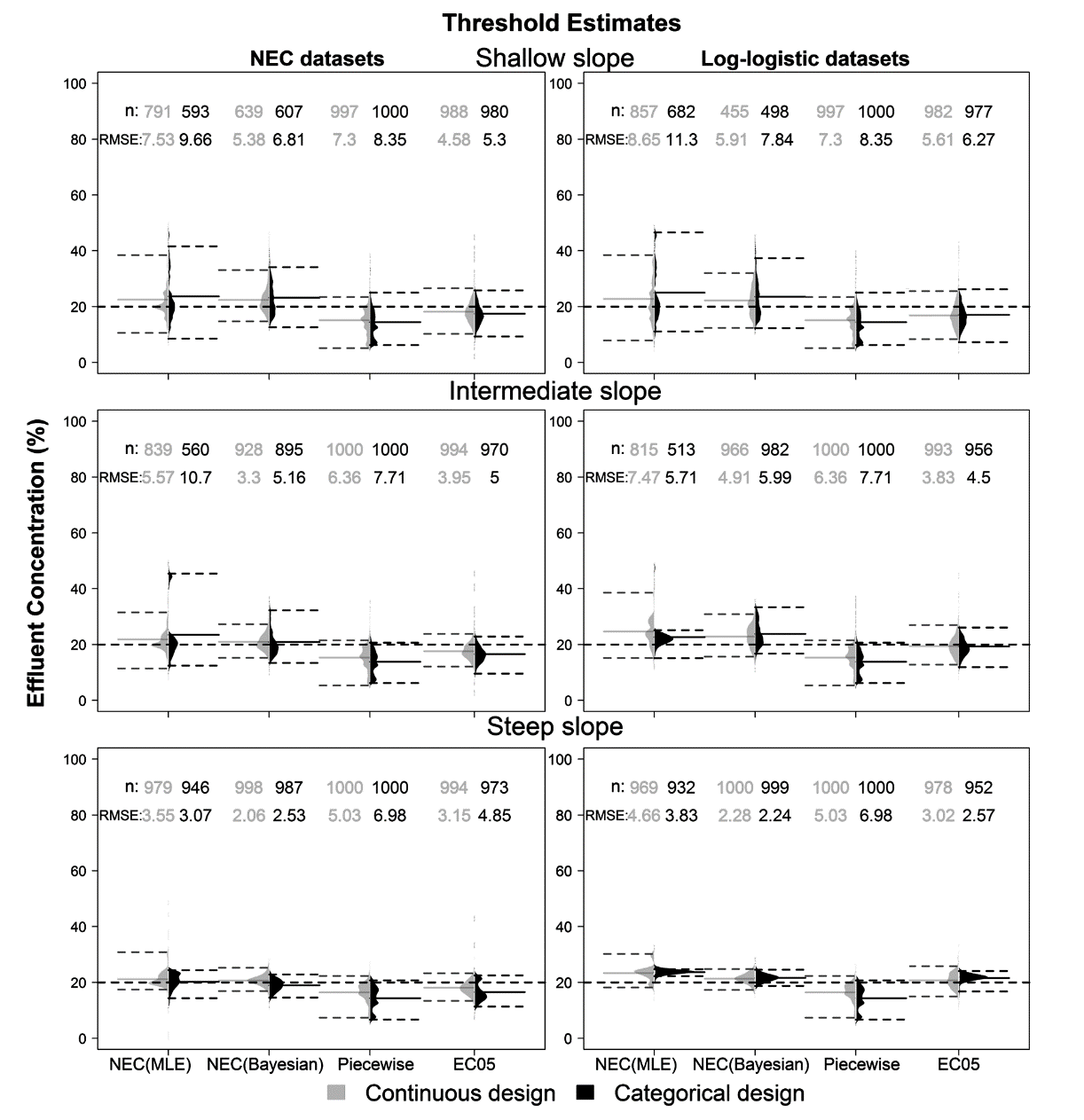
**

**Fig S1. Distribution of the threshold and EC5 estimates for the continuous and categorical designs (with low background mortality) for three different types of curves and for the datasets generated from NEC and log-logistic curves.** Dashed lines represent the true threshold value for the NEC datasets and the apparent threshold in log-logistic datasets, assuming an apparent threshold equal to the NEC models. The numbers above the boxplots are the RMSE of the estimates and the number datasets that the method fitted to the data.

**Fig S2**

**
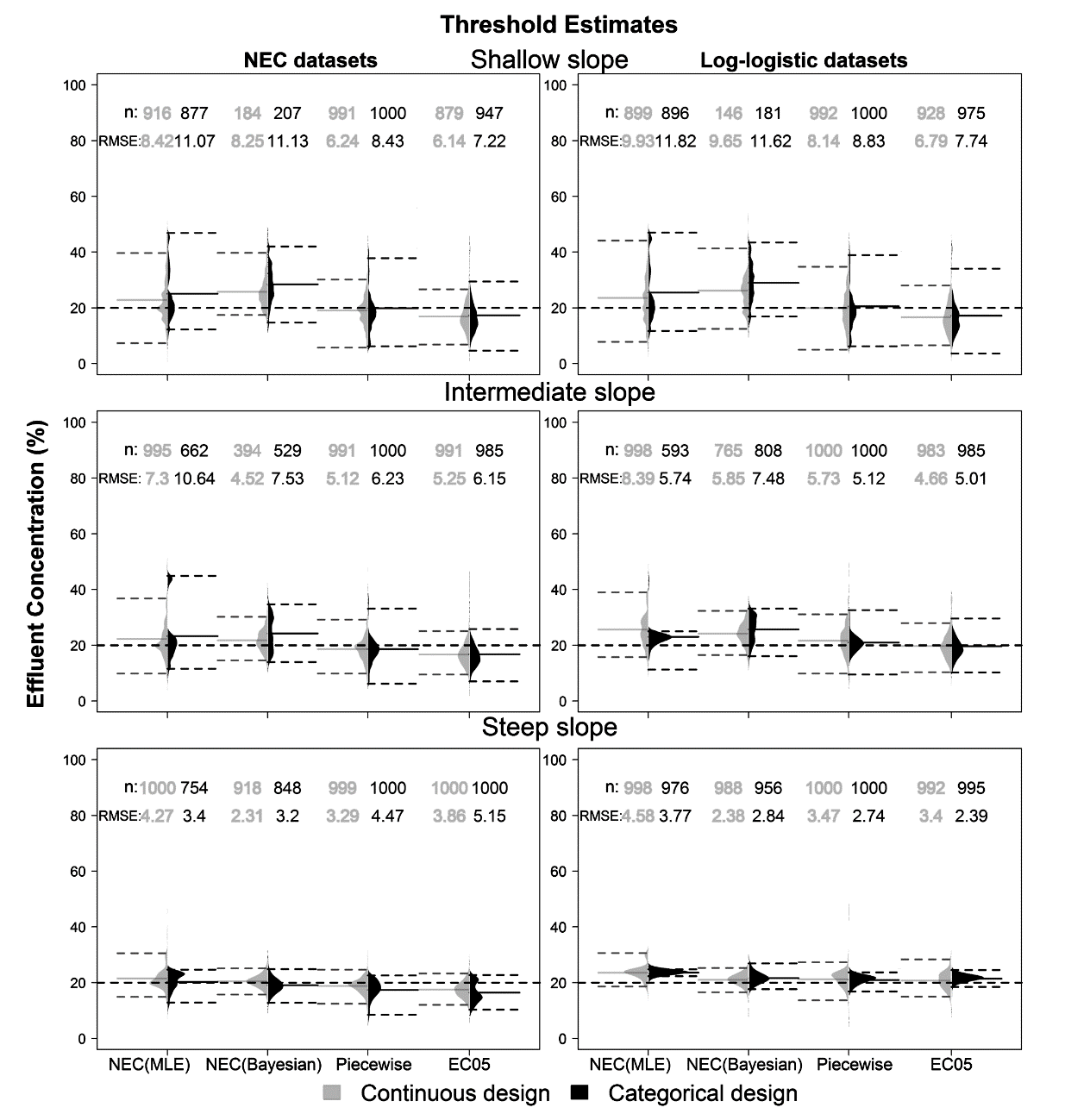
**

**Fig S2. Distribution of the threshold and EC5 estimates for the continuous and categorical designs (with high background mortality) for three different types of curves and for the datasets generated from NEC and log-logistic curves**. Dashed lines represent the true threshold value for the NEC datasets and the apparent threshold in log-logistic datasets, assuming an apparent threshold equal to the NEC models. The numbers above the boxplots are the RMSE of the estimates and the number datasets that the method fitted to the data.
