# Supplementary material for: Comparing statistical analyses to estimate thresholds in ecotoxicology
Source: PLoS One. 2020 Apr 8;15(4):e0231149. doi: 10.1371/journal.pone.0231149 (PMC7141675; doi:10.1371/journal.pone.0231149)
Supplement: S6 Appendix — (DOCX) [file pone.0231149.s006.docx]

**Appendix S6**

**Table S1. Rate of the number of models selected by the AIC and AIC weights between a three-parameter log-logistc and NEC models fitted with maximum likelihood methods.**

|  |  | **Categorical design** | | | | | | | |
| --- | --- | --- | --- | --- | --- | --- | --- | --- | --- |
|  |  | **NEC datasets (%)** | | | | **Log-Logistic datasets (%)** | | | |
| **Slope** | **Background mortality** | **LL** | **NEC** | **W.LL** | **W.NEC** | **LL** | **NEC** | **W.LL** | **W.NEC** |
| **Shallow** | **Low** | 67.7 | 32.3 | 46.0 | 1.1 | 71.8 | 28.2 | 40.4 | 0.5 |
|  | **Medium** | 48.5 | 51.5 | 12.4 | 3.1 | 57.1 | 42.9 | 12.5 | 1.9 |
|  | **High** | 52.0 | 48.0 | 7.3 | 5.0 | 56.6 | 43.4 | 7.7 | 2.0 |
| **Mean:** | | **48.5** | **32.3** | **21.9** | **1.1** | **61.8** | **38.2** | **20.2** | **1.5** |
| **Interm.** | **Low** | 71.5 | 28.5 | 45.6 | 0.2 | 73.3 | 26.7 | 40.6 | 0.7 |
|  | **Medium** | 63.2 | 36.8 | 20.2 | 0.3 | 62.7 | 37.3 | 12.3 | 0.3 |
|  | **High** | 66.8 | 33.2 | 17.5 | 0.7 | 69.7 | 30.3 | 14.5 | 0.1 |
| **Mean:** | | **67.2** | **32.8** | **27.8** | **0.4** | **68.6** | **31.4** | **22.5** | **0.4** |
| **Steep** | **Low** | 49.5 | 50.5 | 4.1 | 2.3 | 81.9 | 18.1 | 6.3 | 3.5 |
|  | **Medium** | 52.5 | 47.5 | 10.4 | 0.8 | 82.4 | 17.6 | 1.1 | 0.7 |
|  | **High** | 63.1 | 36.9 | 22.5 | 0.1 | 84.3 | 15.7 | 2.1 | 0.1 |
| **Mean:** | | **55.0** | **45.0** | **12.3** | **1.1** | **82.9** | **17.1** | **3.2** | **1.4** |
| **Overall mean:** | | **59.4** | **40.6** | **20.7** | **1.5** | **71.1** | **28.9** | **15.3** | **1.1** |
|  |  | **Continuous design** | | | | | | | |
|  |  | **NEC datasets (%)** | | | | **Log-Logistic datasets (%)** | | | |
| **Slope** | **Background mortality** | **LL** | **NEC** | **W.LL** | **W.NEC** | **LL** | **NEC** | **W.LL** | **W.NEC** |
| **Shallow** | **Low** | 43.3 | 56.7 | 25.3 | 1.7 | 61.0 | 39.0 | 22.9 | 1.1 |
|  | **Medium** | 32.2 | 67.8 | 9.3 | 5.9 | 49.2 | 50.8 | 10.4 | 5.6 |
|  | **High** | 33.0 | 67.0 | 9.3 | 10.6 | 50.2 | 49.8 | 11.6 | 6.1 |
| **Mean:** | | **36.2** | **63.8** | **14.6** | **6.1** | **53.5** | **46.5** | **15.0** | **4.3** |
| **Interm.** | **Low** | 38.5 | 61.5 | 20.5 | 3.3 | 64.6 | 35.4 | 28.4 | 0.7 |
|  | **Medium** | 30.8 | 69.2 | 5.2 | 1.7 | 56.1 | 43.9 | 7.3 | 0.6 |
|  | **High** | 34.8 | 65.2 | 6.4 | 1.2 | 54.1 | 45.9 | 6.3 | 1.7 |
| **Mean:** | | **34.7** | **65.3** | **10.7** | **2.1** | **58.3** | **41.7** | **14.0** | **1.0** |
| **Steep** | **Low** | 28.8 | 71.2 | 5.1 | 6.2 | 68.5 | 31.5 | 8.7 | 1.5 |
|  | **Medium** | 27.7 | 72.3 | 2.4 | 2.4 | 67.0 | 33.0 | 3.5 | 0.0 |
|  | **High** | 34.1 | 65.9 | 3.9 | 1.1 | 66.8 | 33.2 | 3.2 | 1.0 |
| **Mean:** | | **30.2** | **69.8** | **3.8** | **3.2** | **67.4** | **32.6** | **5.1** | **0.8** |
| **Overall mean:** | | **33.7** | **66.3** | **9.7** | **3.8** | **59.7** | **40.3** | **11.4** | **2.0** |

**Table S2. Rate of the number of models selected by the DIC and DIC weights between a three-parameter log-logistc and NEC models fitted with MCMC methods.**

|  |  | **Categorical design** | | | | | | | |
| --- | --- | --- | --- | --- | --- | --- | --- | --- | --- |
|  |  | **NEC datasets (%)** | | | | **Log-Logistic datasets (%)** | | | |
| **Slope** | **Background mortality** | **LL** | **NEC** | **W.LL** | **W.NEC** | **LL** | **NEC** | **W.LL** | **W.NEC** |
| **Shallow** | **Low** | 75.0 | 25.0 | 14.5 | 0.1 | 80.4 | 19.6 | 14.1 | 0.3 |
|  | **Medium** | 89.5 | 10.5 | 5.4 | 0.2 | 86.2 | 13.8 | 8.4 | 0 |
|  | **High** | 90.5 | 9.5 | 5.4 | 0.0 | 91.2 | 8.8 | 6.5 | 0.3 |
| **Mean:** | | **85.0** | **15.0** | **8.4** | **0.1** | **85.9** | **14.1** | **9.7** | **0.2** |
| **Interm.** | **Low** | 65.6 | 34.4 | 3.3 | 0.2 | 71 | 29 | 34.2 | 0.7 |
|  | **Medium** | 72.5 | 27.5 | 13.4 | 0.4 | 67.6 | 32.4 | 0.7 | 0.0 |
|  | **High** | 78.3 | 21.7 | 1.9 | 0.0 | 67.1 | 32.9 | 9.4 | 0.0 |
| **Mean:** | | **72.1** | **27.9** | **6.2** | **0.2** | **68.6** | **31.4** | **14.8** | **0.2** |
| **Steep** | **Low** | 27.9 | 72.1 | 0.1 | 0.9 | 8.5 | 91.5 | 0 | 0.0 |
|  | **Medium** | 29.2 | 70.8 | 0.4 | 0.1 | 11.9 | 88.1 | 0.1 | 0.0 |
|  | **High** | 40.3 | 59.7 | 0.8 | 0.0 | 15.9 | 84.1 | 0.2 | 0.0 |
| **Mean:** | | **32.5** | **67.5** | **0.4** | **0.3** | **12.1** | **87.9** | **0.1** | **0.0** |
| **Overall Mean:** | | **63.2** | **36.8** | **5.0** | **0.2** | **55.5** | **44.5** | **8.2** | **0.1** |
|  |  | **Continuous design** | | | | | | | |
|  |  | **NEC datasets (%)** | | | | **Log-Logistic datasets (%)** | | | |
| **Slope** | **Background mortality** | **LL** | **NEC** | **W.LL** | **W.NEC** | **LL** | **NEC** | **W.LL** | **W.NEC** |
| **Shallow** | **Low** | 59 | 41 | 14.6 | 0.2 | 82.5 | 17.5 | 13.6 | 0.1 |
|  | **Medium** | 78.4 | 21.6 | 11.7 | 0.1 | 89.2 | 10.8 | 5.6 | 0.1 |
|  | **High** | 89.5 | 10.5 | 6.3 | 0 | 93.5 | 6.5 | 3.1 | 0 |
| **Mean:** | | **75.6** | **24.4** | **10.9** | **0.1** | **88.4** | **11.6** | **7.4** | **0.1** |
| **Interm.** | **Low** | 39.1 | 60.9 | 2 | 2.2 | 59.7 | 40.3 | 5.8 | 0.6 |
|  | **Medium** | 51.3 | 48.7 | 13.2 | 0.5 | 61.5 | 38.5 | 7.4 | 0.1 |
|  | **High** | 76.9 | 23.1 | 10.6 | 0.1 | 63.7 | 36.3 | 8.6 | 0 |
| **Mean:** | | **55.8** | **44.2** | **8.6** | **0.9** | **61.6** | **38.4** | **7.3** | **0.2** |
| **Steep** | **Low** | 23.9 | 76.1 | 0.7 | 4.9 | 60.3 | 39.7 | 3.9 | 0.4 |
|  | **Medium** | 26.1 | 73.9 | 0.7 | 2.1 | 56.9 | 43.1 | 4.7 | 0 |
|  | **High** | 32.2 | 67.8 | 5.1 | 1.4 | 54.2 | 45.8 | 5.7 | 0.1 |
| **Mean:** | | **27.4** | **72.6** | **2.2** | **2.8** | **57.1** | **42.9** | **4.8** | **0.2** |
| **Overall Mean:** | | **52.9** | **47.1** | **7.2** | **1.3** | **69.1** | **30.9** | **6.5** | **0.2** |

**Table S3. Rate of the number of models selected by the AICc and AICc weights between a two-parameter GLM logit model and a three-parameter GLM logit piecewise regression.**

|  |  | **Categorical design** | | | | | | | |
| --- | --- | --- | --- | --- | --- | --- | --- | --- | --- |
|  |  | **NEC datasets (%)** | | | | **Log-Logistic datasets (%)** | | | |
| **Slope** | **Background mortality** | **LL** | **NEC** | **LL_(w)_** | **NEC_(w)_** | **LL** | **NEC** | **LL_(w)_** | **NEC_(w)_** |
| **Shallow** | **Low** | 55.9 | 44.1 | 0 | 15.1 | 53.5 | 46.5 | 0 | 17.2 |
|  | **Medium** | 32.4 | 67.6 | 0 | 34.3 | 34.7 | 65.3 | 0 | 30.2 |
|  | **High** | 26.9 | 73.1 | 0 | 37.6 | 26.7 | 73.3 | 0 | 41.2 |
| **Mean:** | | **38.4** | **61.6** | **0.0** | **29.0** | **38.3** | **61.7** | **0.0** | **29.5** |
| **Interm.** | **Low** | 46.3 | 53.7 | 0 | 20.7 | 26 | 74 | 0 | 43.6 |
|  | **Medium** | 22.9 | 77.1 | 0 | 46.7 | 9.3 | 90.7 | 0 | 74 |
|  | **High** | 15.5 | 84.5 | 0 | 56.7 | 4.8 | 95.2 | 0 | 82.2 |
| **Mean:** | | **28.2** | **71.8** | **0.0** | **41.4** | **13.4** | **86.6** | **0.0** | **66.6** |
| **Steep** | **Low** | 33.2 | 66.8 | 0 | 36.5 | 32.2 | 67.8 | 0 | 25 |
|  | **Medium** | 15.6 | 84.4 | 0 | 59.1 | 16 | 84 | 0.1 | 46.3 |
|  | **High** | 10.9 | 89.1 | 0 | 66.9 | 12.6 | 87.4 | 0.6 | 51.4 |
|  | **Mean:** | **19.9** | **80.1** | **0.0** | **54.2** | **20.3** | **79.7** | **0.2** | **40.9** |
| **Overall Mean:** | | **28.8** | **71.2** | **0.0** | **41.5** | **24.0** | **76.0** | **0.1** | **45.7** |
|  |  | **Continuous design** | | | | | | | |
|  |  | **NEC datasets (%)** | | | | **Log-Logistic datasets (%)** | | | |
| **Slope** | **Background mortality** | **LL** | **NEC** | **LL_(w)_** | **NEC_(w)_** | **LL** | **NEC** | **LL_(w)_** | **NEC_(w)_** |
| **Shallow** | **Low** | 38.4 | 61.6 | 0 | 27.8 | 45 | 55 | 0 | 22.1 |
|  | **Medium** | 21.5 | 78.5 | 0 | 46.3 | 27.5 | 72.5 | 0.1 | 40.9 |
|  | **High** | 17.1 | 82.9 | 0.2 | 51 | 19 | 81 | 0 | 50.1 |
|  | **Mean:** | **25.7** | **74.3** | **0.1** | **41.7** | **30.5** | **69.5** | **0.0** | **37.7** |
| **Interm.** | **Low** | 27.2 | 72.8 | 0 | 46.4 | 20.9 | 79.1 | 0 | 56.2 |
|  | **Medium** | 13.2 | 86.8 | 0 | 66.7 | 6.4 | 93.6 | 0 | 77.8 |
|  | **High** | 8.4 | 91.6 | 0 | 70.7 | 3.7 | 96.3 | 0 | 86.3 |
|  | **Mean:** | **16.3** | **83.7** | **0.0** | **61.3** | **10.3** | **89.7** | **0.0** | **73.4** |
| **Steep** | **Low** | 15.5 | 84.5 | 0 | 65.4 | 15.3 | 84.7 | 0 | 59 |
|  | **Medium** | 4.5 | 95.5 | 0 | 85.4 | 7.7 | 92.3 | 0 | 73.9 |
|  | **High** | 1.8 | 98.2 | 0 | 91.4 | 0.6 | 99.4 | 0 | 96.8 |
|  | **Mean:** | **7.3** | **92.7** | **0.0** | **80.7** | **7.9** | **92.1** | **0.0** | **76.6** |
| **Overall Mean:** | | **16.4** | **83.6** | **0.0** | **61.2** | **16.2** | **83.8** | **0.0** | **62.6** |
